# Trained immunity of alveolar macrophages requires metabolic rewiring and type 1 interferon signaling

**DOI:** 10.1038/s41385-022-00528-5

**Published:** 2022-07-18

**Authors:** Sophie Zahalka, Philipp Starkl, Martin L. Watzenboeck, Asma Farhat, Mariem Radhouani, Florian Deckert, Anastasiya Hladik, Karin Lakovits, Felicitas Oberndorfer, Caroline Lassnig, Birgit Strobl, Kristaps Klavins, Mai Matsushita, David E. Sanin, Katarzyna M. Grzes, Edward J. Pearce, Anna-Dorothea Gorki, Sylvia Knapp

**Affiliations:** 1 Research Laboratory of Infection Biology, Department of Medicine I, Medical University of Vienna, Vienna, Austria; 2 CeMM, Research Center for Molecular Medicine of the Austrian Academy of Sciences, Vienna, Austria; 3 Department of Pathology, Medical University of Vienna, Vienna, Austria; 4 Institute of Animal Breeding and Genetics, Department of Biomedical Sciences, University of Veterinary Medicine Vienna, Vienna, Austria; 5 Biomodels Austria, Department of Biomedical Sciences, University of Veterinary Medicine Vienna, Vienna, Austria; 6 Institute of General Chemical Engineering, Riga Technical University, Riga, Latvia; 7 Department of Immunometabolism, Max Planck Institute of Immunobiology and Epigenetics, Freiburg, Germany; 8 The Bloomberg-Kimmel Institute for Cancer Immunotherapy at Johns Hopkins, Johns Hopkins University, Baltimore, MD USA

## Abstract

Environmental microbial triggers shape the development and functionality of the immune system. Alveolar macrophages (AMs), tissue-resident macrophages of the lungs, are in constant and direct contact with inhaled particles and microbes. Such exposures likely impact AM reactivity to subsequent challenges by immunological imprinting mechanisms referred to as trained immunity. Here, we investigated whether a ubiquitous microbial compound has the potential to induce AM training in vivo. We discovered that intranasal exposure to ambient amounts of lipopolysaccharide (LPS) induced a pronounced AM memory response, characterized by enhanced reactivity upon pneumococcal challenge. Exploring the mechanistic basis of AM training, we identified a critical role of type 1 interferon signaling and found that inhibition of fatty acid oxidation and glutaminolysis significantly attenuated the training effect. Notably, adoptive transfer of trained AMs resulted in increased bacterial loads and tissue damage upon subsequent pneumococcal infection. In contrast, intranasal pre-exposure to LPS promoted bacterial clearance, highlighting the complexity of stimulus-induced immune responses, which likely involve multiple cell types and may depend on the local immunological and metabolic environment. Collectively, our findings demonstrate the profound impact of ambient microbial exposure on pulmonary immune memory and reveal tissue-specific features of trained immunity.

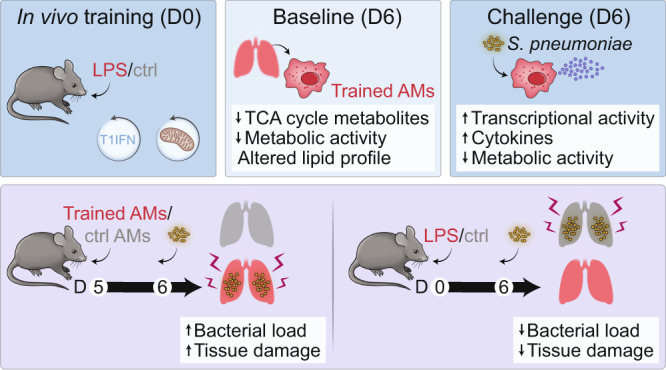

## Introduction

Our immune system is invariably shaped by microbial encounters and exposure to environmental pathogen-associated molecular patterns (PAMPs). Such interactions can result in long-term functional changes of innate immune cells, which enable an increased responsiveness to secondary challenges^1^. This phenomenon, referred to as trained immunity, has broadened our understanding of innate immunity and represents a critical component of immune cell memory^1^. Trained immunity can be induced by endogenous or exogenous compounds (e.g. β-glucan, oxLDL, cytokines) and has been described for a wide range of cell types, including monocytes, macrophages and NK cells, as well as hematopoietic stem cells and multipotent progenitor cells^2^. While the molecular basis of trained immunity is not yet fully understood, it is known that innate memory responses mechanistically depend on a complex interplay between epigenetic regulation and cellular metabolism^3^.

The lungs are continually exposed to particles and microbes from the external environment^4^, which may impact local immune responses and induce innate memory. Lipopolysaccharide (LPS), the major component of Gram-negative bacterial cell walls, is a ubiquitously present PAMP that can be detected in airborne particles, such as organic dust and cigarette smoke^5^, and initiates a proinflammatory response upon recognition by the innate immune system^6^. There are considerable variations in the amount of personal, ambient LPS exposure. Urban LPS concentrations are generally below 10 inhalable endotoxin units (EU)/m^3^^7^, whereas ranges of 300–6600 EU/m^3^ have been reported for endotoxin-rich environments, such as livestock farming^8^, corresponding to 0.15–3.3 EU per breath (assuming 0.5 L human tidal volume^9^). While the consequences of LPS inhalation are diverse and complex, recent epidemiological studies have demonstrated a protective effect of environmental endotoxin exposure on the development of allergic diseases^10^.

Being located at the interface of the airways and the environment, alveolar macrophages (AMs) constitute the first line of innate cellular defense against inhaled microbes^11^ and are in direct contact with airborne allergens, environmental agents and PAMPs, including LPS. Given their unique location, AMs exhibit a distinctive cellular profile that tightly regulates their activation state to avoid excessive inflammatory responses^12^. As such, they display limited plasticity and exhibit only moderate transcriptional and functional changes following severe insults such as bleomycin-induced fibrosis or influenza infection^13^. Furthermore, AMs are metabolically adapted to the low glucose levels of the alveolar space and depend on oxidative phosphorylation (OXPHOS) while maintaining only minimal glycolytic activity in homeostatic and inflammatory conditions^14^. Despite their hyporesponsive state, AMs are key players in the innate pulmonary defense during respiratory infections and eliminate invading pathogens by processes such as phagocytosis or secretion of antimicrobial peptides, while playing an equally important role in the restoration of homeostasis^11^. AM-mediated initiation and resolution of inflammation have been shown to be particularly relevant during bacterial pneumonia, which is most commonly caused by the Gram-positive bacterium *Streptococcus pneumoniae (S. pneumoniae)*^15^. Due to their unique cellular properties, AMs are interesting candidates to study tissue-specific aspects of trained immunity. Yet, our current knowledge about innate memory responses of AMs and the underlying cellular mechanisms is limited.

In this study, we investigated whether exposure to ambient amounts of LPS can modulate AM function by inducing trained immunity. We applied a combination of genetic, epigenetic and metabolic analyses to uncover the unique cellular properties of AM memory and assessed the consequences of LPS training in the context of pneumococcal infection.

## Results

### LPS exposure induces trained immunity in alveolar macrophages

**Fig. 1:**
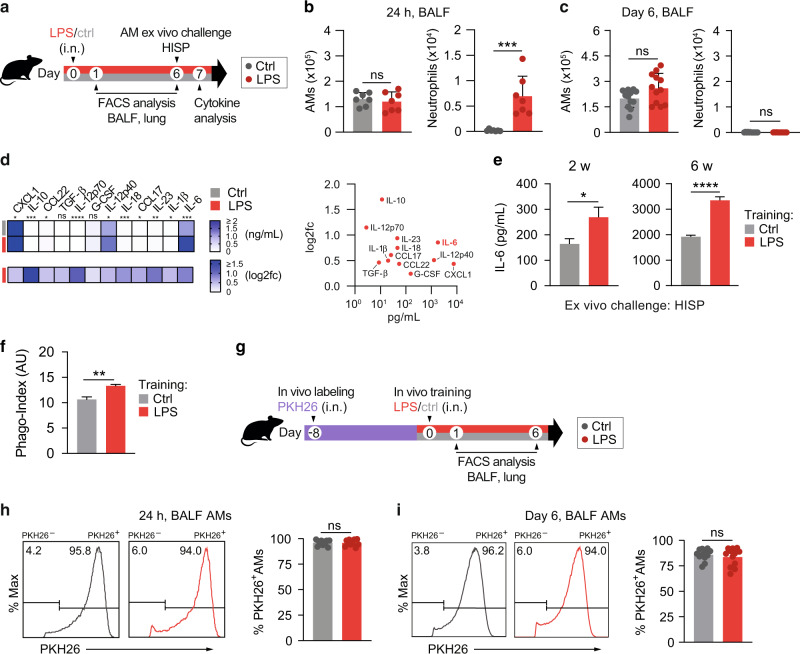
LPS exposure induces trained immunity in alveolar macrophages. **a** Experimental setup for i.n. LPS (1 ng/mouse) or saline exposure, followed by FACS analysis (BALF, lung) or AM cytokine analysis upon ex vivo bacterial challenge. **b**, **c ** Absolute numbers of BALF AMs and neutrophils, measured by flow cytometry 24 h (**b**) and six days (**c**) after in vivo treatment. **d** LEGENDplex analysis of LPS-exposed and control AMs upon ex vivo HISP-challenge (16 h). Absolute cytokine levels (top heat map) and log_2_ fold change (log_2_fc) of LPS-exposed AMs versus means of control AMs (bottom heat map) are shown. The right panel compares absolute levels and log_2_fc of trained AMs. **e** IL-6 levels of LPS-exposed and control AMs upon ex vivo bacterial challenge two or six weeks after treatment. **f** Phagocytosis index (AU: arbitrary units) of trained and control AMs, isolated on day six after in vivo training, followed by ex vivo stimulation with FITC-labeled HISP and FACS analysis. **g** Experimental setup for PKH26 labeling eight days prior to in vivo training. **h**, **i** Representative histograms of PKH26 MFI (gated on CD11c^+^ Siglec F^+^ AMs) and percentage of PKH26^+^ AMs 24 h (**h**) and six days (**i**) after training. Graphs show means + SD of 7 (**b**) or 11–12 (**c**, **h**, **i**) biological replicates, means of 7–8 biological replicates (**d**) or means + SD of 3–5 technical replicates (**e**, **f**). Data (**b**, **c**, **e**, **f**) are representative of two independent experiments. Statistical analysis: student’s *t*-test. ns, not significant. *p ≤ 0.05, **p ≤ 0.01, ***p ≤ 0.001, ****p ≤ 0.0001.

The respiratory tract is continuously exposed to airborne microbial products, which modulate the pulmonary immune system. Due to their strategic location in the alveoli of the lungs, AMs are in direct contact with inhaled particles and microbes, and thus represent potential candidates to develop trained immunity. To investigate whether ambient concentrations of ubiquitous airborne compounds can elicit AM memory, we administered 1 ng LPS (∼0.5 EU/mouse; an amount in relation potentially inhaled by humans^8^) or endotoxin-free saline intranasally (i.n.) to wild type C57BL/6 J mice (Fig. 1a). This treatment induced an acute inflammatory response, characterized by a transient influx of neutrophils after 24 h (Fig. 1b). Six days later, neutrophils were no longer detectable in the BAL fluid (BALF; Fig. 1c), and post-lavage lung immune cell numbers were comparable to the control group, except for dendritic cell and B cell numbers, which remained moderately elevated in LPS-exposed lungs (Fig. S1a–c). These data support the transient nature of LPS-triggered effects, and exclude overt signs of persisting lung inflammation. To assess whether LPS exposure modulates AM immunity, we isolated AMs by BAL six days after i.n. LPS treatment and challenged the cells ex vivo with heat-inactivated *S. pneumoniae* (HISP, Fig. 1a). LPS-exposed AMs produced higher amounts of multiple cytokines and chemokines, including C-X-C Motif Chemokine Ligand (CXCL)-1, interleukin (IL)-1β, IL-10, IL-12p40 and IL-6 compared to control (saline-exposed) AMs (Fig. 1d, Fig. S2a), indicating an innate memory effect. Taking absolute concentrations and log_2_ fold changes into account (Fig. 1d), we selected IL-6, a cytokine critically involved in host immunity^16^, as the most stable readout and decided to use it as a surrogate for LPS-induced AM memory in subsequent experiments. In support of this choice, we continued to observe elevated IL-6 responses by AMs two and six weeks after intranasal LPS treatment (Fig. 1e), indicating long-lasting cellular reprogramming following ambient LPS exposure.

Given that phagocytosis and efferocytosis are key effector functions of AMs, we next decided to investigate whether these processes are modulated six days after in vivo training. To analyze AM-mediated phagocytosis, AMs were isolated by BAL and incubated with FITC-labeled HISP, followed by FACS analysis. These experiments revealed that the phagocytic capacity of trained AMs was enhanced in comparison to saline-treated controls (Fig. 1f). AM-mediated efferocytosis was assessed after intratracheal transfer of CFSE-labeled apoptotic thymocytes on day six after training (Fig. S2b) and did not reveal any differences between the groups (Fig. S2c, d). However, we noticed that LPS-exposed AMs showed elevated surface expression of MerTK (Fig. S2e) and Axl (Fig. S2f), two TAM family receptors known to promote apoptotic cell removal^17^.

AMs are predominantly of embryonic origin and self-maintain locally with minimal contribution of circulating monocytes under steady-state conditions^18^. However, upon infection or severe lung injury, the alveolar niche can be replenished by monocytes, which get recruited to the lungs and acquire an AM profile under the influence of the local microenvironment^13^. In order to determine whether the transient inflammatory response to LPS inhalation induced replenishment of tissue-resident AMs by monocytes, we labeled resident AMs by i.n. administration of the fluorescent dye PKH26^19^ eight days prior to LPS treatment (Fig. 1g). Frequencies of PKH26^+^ and PKH26^−^ AMs were determined 24 h and six days after training. The dye selectively labeled lung-resident CD11c^+^ Siglec F^+^ AMs while CD11b^+^ Ly6C^+^ monocytes remained PKH26-negative (Fig. S2g). Of note, the percentage of PKH26^+^ CD11c^+^ Siglec F^+^ AMs in BALF (Fig. 1h, i) and post-lavage lung tissue (Fig. S2h) was comparable between LPS-treated and control mice at both timepoints investigated. This indicates that the resident AM population was not replenished by inflammatory monocytes upon in vivo training. In conclusion, we showed that i.n. LPS exposure trains the local AM pool for increased cytokine production and phagocytosis following secondary bacterial challenge.

### AM training depends on type 1 interferon signaling

LPS-mediated signaling is accompanied by the production of type 1 interferons (IFNs; e.g. IFN-β)^20^ and type 2 IFNs (IFN-γ)^21^, which have the capacity to modulate immune responses. Yao et al. reported that IFN-γ, produced by T-cells during respiratory adenoviral infection, primes AMs for enhanced immune activity upon secondary pneumococcal challenge^22^. To test whether IFN-γ- or T cell-mediated responses contribute to LPS-induced AM memory, we applied our training model (in vivo training, followed by ex vivo AM challenge) to IFN-γ-receptor-deficient (Ifngr1^−/−^) and Rag2-deficient (Rag2^−/−^) mice, respectively. These experiments showed that the enhanced IL-6 response of LPS-exposed AMs occurred independently of IFN-γ-receptor signaling (Fig. S3a) and adaptive immunity (Fig. S3b).

**Fig. 2:**
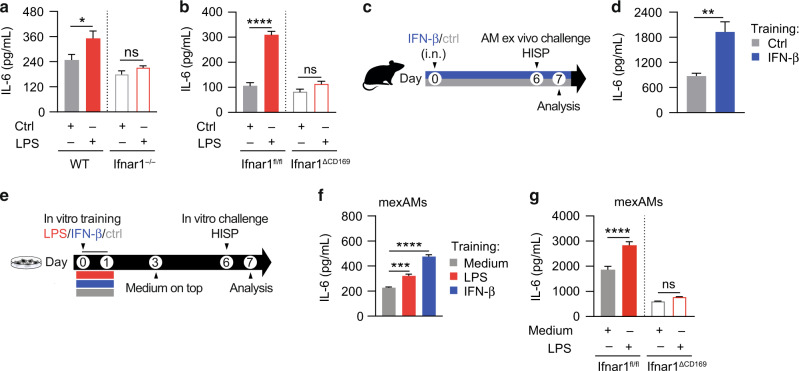
LPS-induced AM training is driven by type 1 interferon signaling. **a**, **b** IL-6 levels of HISP-challenged (16 h) LPS-trained and control AMs, isolated six days after in vivo training (performed as described in Fig. 1) of Ifnar1^−/−^ and WT control mice (**a**) or Ifnar1^ΔCD169^ and Ifnar1^fl/fl^ control mice (**b**). **c** Experimental setup for i.n. treatment with IFN-β (2000 U/mouse) or saline (control). AMs were isolated on day six, followed by ex vivo challenge with HISP (16 h). **d** IL-6 levels of IFN-β-trained and control AMs after ex vivo restimulation. **e** Experimental setup for in vitro LPS (10 ng/mL)- or IFN-β (800 U/ mL)- training of mexAMs, followed by in vitro HISP challenge (16 h) six days later. **f** IL-6 levels of control, LPS-trained and IFN-β-trained WT mexAMs after HISP challenge. **g** IL-6 levels of LPS-trained and control Ifnar1^ΔCD169^ and Ifnar1^fl/fl^ mexAMs after HISP challenge. Graphs show means + SEM of 4-5 technical replicates. In (**a**–**d**), four biological replicates per group were pooled and seeded as technical replicates for stimulation. Data are representative of two independent experiments. Statistical analysis: two-way ANOVA (factor 1: training; factor 2: genotype) (**a**, **b**, **g**), student’s *t*-test (**d**) or one-way ANOVA (**f**). ns, not significant. *p ≤ 0.05, **p ≤ 0.01, ***p ≤ 0.001, ****p ≤ 0.0001.

LPS is a potent inducer of type 1 IFNs, which can in turn potentiate LPS-mediated immune responses^23^. To investigate whether type 1 IFN signaling plays a role in LPS-induced AM memory, we trained type 1 IFN-receptor-deficient (Ifnar1^−/−^) and control mice, and analyzed AM IL-6 production upon ex vivo HISP challenge six days later. Interestingly, AMs retrieved from LPS-exposed Ifnar1^−/−^ mice were unable to mount a trained response (Fig. 2a), suggesting that type 1 IFN signaling plays an important role in the establishment of AM memory. Of note, this effect was not restricted to IL-6, as IL-12p40 and IL-12p70 responses were similarly diminished upon Ifnar1 deficiency (Fig. S3c, d).

Based on these findings, we aimed to dissect whether type 1 IFNs promote AM training in a direct or indirect manner. Using Ifnar1^ΔCD169^ and Ifnar1^fl/fl^ control mice, we found that macrophage-specific deficiency of type 1 IFN receptor expression abolished AM memory (Fig. 2b), indicating that AMs need to directly sense type 1 IFNs during LPS-mediated training. This prompted us to investigate whether local administration of type 1 IFNs has the potential to induce trained immunity in AMs. For this purpose, we treated wild type mice i.n. with IFN-β or saline and examined the responsiveness of AMs six days later (Fig. 2c). Similar to LPS-training, IFN-β exposure increased the IL-6 production by AMs upon secondary, bacterial challenge, indicating an innate memory effect (Fig. 2d).

Given that type 1 IFNs act downstream of TLR4 signaling and can affect cellular immunity via autocrine signaling^24^, we went on to explore whether AM memory can be induced in a cell-autonomous manner and generated murine ex vivo cultured AMs (mexAMs)^25^ from primary wild type AMs. Following in vitro expansion, mexAMs were stimulated for 24 h with LPS, IFN-β or medium, allowed to rest for five days, and subsequently challenged with HISP on day six after training (Fig. 2e). Similar to in vivo AM training, LPS- or IFN-β-exposed mexAMs produced increased amounts of IL-6 upon bacterial challenge (Fig. 2f), indicating that these stimuli have the potential to *directly* induce AM memory. Applying the same regimen to Ifnar1^ΔCD169^ and Ifnar1^fl/fl^ control mexAMs, we could further demonstrate that autocrine type 1 IFN signaling can mediate LPS-induced in vitro training (Fig. 2g).

Collectively, our data suggest that type 1 IFNs play an important role in the establishment of LPS-mediated AM memory.

### Trained AMs exhibit an altered transcriptional profile upon secondary bacterial challenge

**Fig. 3:**
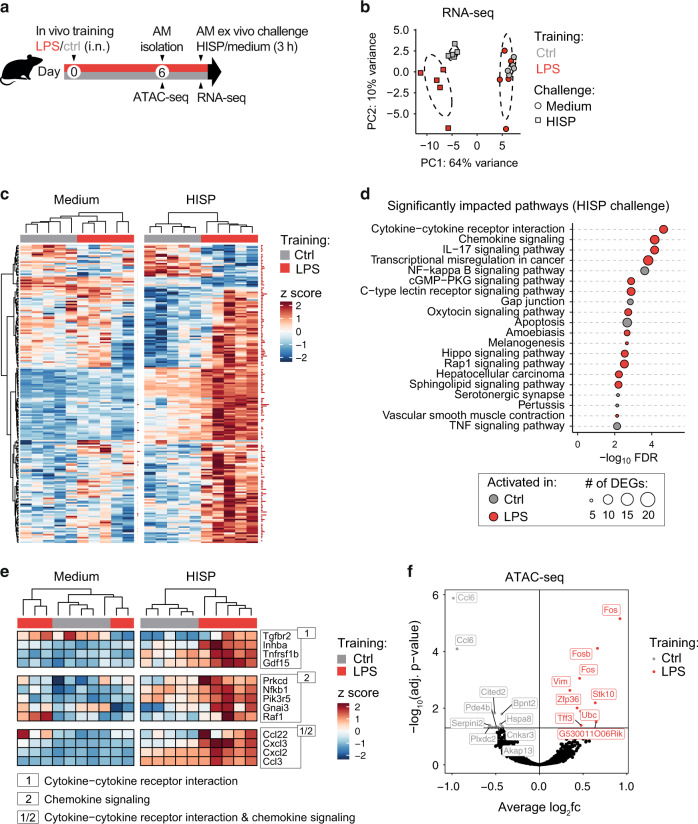
LPS-trained AMs display an altered gene expression profile upon ex vivo challenge. **a** Experimental setup for ATAC-seq and RNA-seq analysis of AMs isolated on day six after in vivo training. **b** Principal component analysis of normalized gene expression data obtained from in vivo trained AMs upon 3 h ex vivo HISP challenge (squares) or medium (circles). **c** Heatmap depicting DEGs in trained and control AMs upon medium or HISP challenge. Samples are clustered by unsupervised clustering. Data are *rlog* transformed, followed by z-score scaling. Cutoff: adjusted *p*-value ≤ 0.1; red horizontal bars adjacent to heatmaps indicate statistical significance. **d** Top 20 KEGG pathways in HISP-challenged trained and control AMs. Circle size indicates the number of DEGs associated with the respective pathway. **e** Heatmaps depicting DEGs identified in the top two differentially regulated pathways in trained and control AMs upon medium or HISP challenge. Data are *rlog* transformed, followed by z-score scaling. **f** Volcano plot displaying differentially accessible regions (DARs; padj ≤ 0.05) of trained versus control AMs identified by ATAC-seq analysis six days after in vivo training. Labels indicate top 10 DARs per group.

Trained immunity is defined as the altered reactivity to a secondary trigger induced by prior exposure to a training stimulus^26^. In contrast to primed immune responses, this phenomenon is characterized by the return to a baseline state after initial activation^26^. Mechanistically, trained immunity has been associated with epigenetic remodeling and metabolic reprogramming, two processes that serve as the molecular basis for altered gene expression upon secondary challenge^3^. To identify transcriptional changes of LPS-exposed and control AMs at baseline and upon subsequent bacterial stimulation, AMs were isolated six days after training and incubated for 3 h with medium or HISP (Fig. 3a). Principal component analysis (PCA) of RNA-seq results revealed a high similarity between trained and control AMs (Fig. 3b, c), and only 10 differentially expressed genes (DEGs) at baseline (Table S1). In contrast, transcriptional profiles clustered according to the preceding training stimulus upon bacterial challenge (Fig. 3b, c), which correlated with 165 upregulated and 27 downregulated genes identified in LPS-trained compared to control AMs (Table S2). DEGs detected upon HISP challenge mapped to different Kyoto Encyclopedia of Genes and Genomes (KEGG) pathways (Fig. 3d), with “cytokine-cytokine receptor interaction“ and “chemokine signaling“ being most differentially regulated. Among these pathways, signaling-related genes (*Prkcd, Nfkb1, Raf1*) as well as genes encoding chemokines of CC (*Ccl22, Ccl3*) and CXC (*Cxcl2, Cxcl3*) subfamilies were differentially expressed upon HISP stimulation (Fig. 3e). In accordance, CXCL1 protein levels were markedly increased upon ex vivo challenge of trained AMs (Fig. S2a).

To assess whether the altered transcriptional responsiveness of LPS-exposed AMs was associated with persistent epigenetic changes reflected by altered chromatin accessibility, BAL AMs were processed for Assay for Transposase-Accessible Chromatin (ATAC)-seq analysis on day six after in vivo training (Fig. 3a). In total, we identified 24 differentially accessible regions (DARs; Fig. S4a and Table S3; FDR ≤ 0.05), nine of which were more accessible in the trained group (Fig. 3f). Among these, three DARs were annotated to the genes *Fos* (2 DARs) or *Fosb* (1 DAR), which are associated with transcriptional regulation of multiple biological processes, including cell migration, differentiation and inflammation^27^.

Next we considered the possibility that accelerated gene expression upon secondary challenge may result from altered baseline deposition of permissive histone marks, such as H3K4 methylation or H3K27 acetylation^28^. To test whether AM training depends on methylation- or acetylation events established during LPS exposure, we trained WT mexAMs in presence of the methyltransferase inhibitor 5’-deoxy-5′-methylthioadenosine^29^ (MTA), the acetyltransferase inhibitor anacardic acid^30^ or DMSO. After 24 h, cells were washed and allowed to rest in medium until bacterial challenge (Fig. S4b). Neither of the inhibitors influenced the trained IL-6 response (Fig. S4c), suggesting that the targeted epigenetic enzymes do not contribute to LPS-induced AM memory.

Collectively, trained and control AMs displayed similar gene expression levels and few changes in chromatin accessibility at baseline, but mounted an augmented transcriptional response upon secondary, bacterial challenge.

### Secondary metabolic AM responses are modulated by prior LPS exposure

**Fig. 4:**
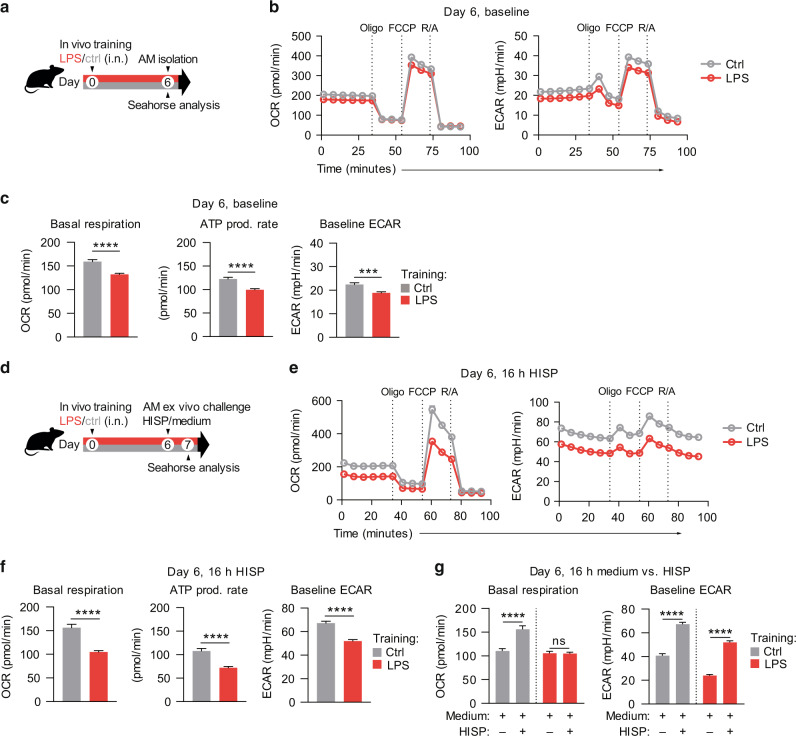
LPS exposure persistently alters the metabolic state and responsiveness of AMs. **a** Experimental setup for Seahorse analyses of AMs on day six after in vivo training with LPS/saline. **b** OCR and ECAR of trained and control AMs, measured at baseline and after sequential treatment with oligomycin (Oligo), FCCP and rotenone/antimycin A (R/A). **c** Quantification of baseline OCR (basal respiration), ATP production rate and baseline ECAR. **d** Experimental setup for Seahorse analyses of AMs upon ex vivo HISP or medium challenge (16 h) on day six after training. **e** OCR and ECAR of trained and control AMs, 16 h after HISP stimulation, measured at baseline and after sequential treatment with indicated drugs. **f** Quantification of basal respiration, ATP production rate and baseline ECAR 16 h after HISP challenge. **g** Quantification of baseline OCR and ECAR 16 h after stimulation with HISP in medium versus medium only. Graphs show means + SEM of 10-11 technical replicates from pooled biological replicates (*n* = 5-8). Data are representative of two independent experiments. Statistical analysis: student’s *t*-test. ns, not significant. ***p ≤ 0.001, ****p ≤ 0.0001; OCR, oxygen consumption rate; ECAR, extracellular acidification rate; FCCP, carbonyl cyanide-4-(trifluoromethoxy)phenylhydrazone.

Recent research highlighted the critical impact of cellular metabolism on the functional state of immune cells, including the induction, maintenance and regulation of trained immunity^31^. Importantly, metabolic pathways do not only provide energy and macromolecular building blocks, but can directly influence the epigenetic machinery by generating intermediate metabolites that serve as substrates or cofactors^32^. In monocytes and macrophages, glycolysis, glutaminolysis and cholesterol synthesis have been described to play crucial roles in the induction of trained immunity, with increased glycolysis defined as hallmark of trained macrophages^31,32^. AMs, however, are unique tissue-resident immune cells that are metabolically adapted to the remarkably low glucose concentration of the alveolar space, and primarily utilize OXPHOS to meet their energy demands^14^. We therefore speculated that the metabolic characteristics of trained AMs may differ from those classically associated with trained monocytes and macrophages. To investigate the baseline metabolism of AMs six days after in vivo training, we performed Seahorse analyses (Fig. 4a, b). Compared to control AMs, LPS-exposed AMs displayed a reduced basal metabolic activity, reflected by a decreased oxygen consumption rate (OCR) and extracellular acidification rate (ECAR) (Fig. 4c). Sequential inhibition of selected electron transport chain (ETC) components further revealed a reduced ATP production rate of trained AMs (Fig. 4c), while maximum respiratory capacity and spare respiratory capacity (SRC) were unaltered (Fig. S5a). To assess how prior LPS exposure affects AM metabolism upon secondary, bacterial challenge, we performed Seahorse analyses of trained and control AMs 16 h after incubation with HISP (Fig. 4d, e). Interestingly, basal OCR and ECAR, ATP production (Fig. 4f), as well as maximum and spare respiratory capacity (Fig. S5b) were significantly decreased in trained cells. While saline-exposed AMs exhibited an increased OCR upon bacterial stimulation (16 h; compared to incubation with medium only), trained AMs displayed similar OCR levels in presence or absence of HISP (Fig. 4g). Although both trained and control cells increased their ECAR upon bacterial challenge (Fig. 4g), the absolute ECAR of trained AMs was lower than that of control AMs at the investigated time point.

In summary, these experiments demonstrate that ambient LPS exposure rewires AM metabolism, which further impacts the metabolic response to subsequent bacterial challenge.

### LPS training induces changes in AM metabolite and lipid composition

**Fig. 5:**
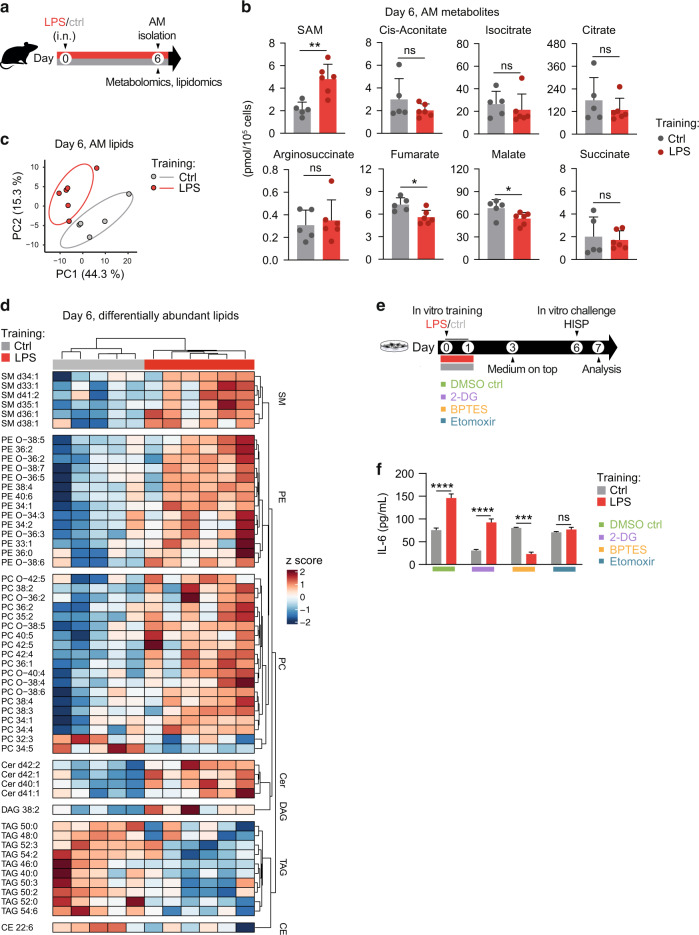
LPS-induced metabolic activation contributes to the establishment of AM memory. **a** Experimental setup for metabolomic/lipidomic analyses of AMs on day six after in vivo training with LPS/saline. **b** AM intracellular metabolites related to the tricarboxylic acid (TCA) cycle. SAM: S-adenosyl-methionine. **c** Principal component analysis of centered log-ratio transformed AM lipidomics data. **d** Heatmap displaying differentially abundant lipids (FDR 0.2, *p*-value ≤ 0.05) of trained and control AMs. Metabolomic/lipidomic analyses were performed with 5-6 biological replicates per group. Cer ceramides; DAG diacylglycerols; PE phosphatidylethanolamines; SM sphingomyelins; PC phosphatidylcholines; CE cholesterol esters; TAG triacylglycerols. **e** Experimental setup for mexAM training with LPS or medium in presence of indicated metabolic inhibitors or DMSO, followed by in vitro HISP challenge (16 h) six days later. **f** IL-6 levels of mexAMs stimulated as described in **e**. Data are representative of two independent experiments. Graphs show means + SD of 5-6 biological replicates (**b**) or means + SEM of 4-5 technical replicates (**f**). Statistical analysis: student’s *t*-test (**b**) and two-way ANOVA (**f**) (factor 1: training; factor 2: inhibitor). ns, not significant. *p ≤ 0.05, **p ≤ 0.01, ***p ≤ 0.001, ****p ≤ 0.0001.

Based on these findings, we went on to investigate the effects of LPS training on the metabolite and lipid composition of AMs. CD11c^+^ Siglec F^+^ AMs were FACS-sorted six days after LPS administration and subjected to LC-MS/MS-based analyses (Fig. 5a; Fig. S6a). Targeted metabolomic analysis revealed that trained AMs contained increased amounts of S-adenosyl-methionine (SAM; Fig. 5b), an essential metabolite synthesized from methionine and ATP^33^. In accordance, intracellular amino acid profiles (Fig. S6b) showed a trend for increased methionine concentrations in trained AMs compared to control cells (Fig. S6c). SAM acts as a universal methyl group donor for RNA, DNA, lipids and proteins and was reported to drive a proinflammatory macrophage phenotype in the context of LPS-induced inflammation^34^. In addition, LPS exposure modulated metabolites of the tricarboxylic acid (TCA) cycle, with fumarate and malate being significantly reduced compared to control AMs (Fig. 5b). Interestingly, lipidomic analysis identified profound differences between LPS- and saline-exposed AMs on day six after training (Fig. 5c). Trained AMs contained substantially higher amounts of selected ceramides (Cer), phosphatidylethanolamines (PE), sphingomyelins (SM) and phosphatidylcholines (PC), essential membrane lipids that can directly or indirectly impact membrane receptor signaling^35^. In contrast, triacylglycerol (TAG) levels were strongly reduced in trained compared to control AMs (Fig. 5d). TAGs serve as cellular energy stores that fuel cell-intrinsic ATP production in the mitochondria by providing free fatty acids for β-oxidation^36^. In summary, these findings imply that in vivo LPS exposure profoundly modulates the metabolite and lipid composition of AMs.

### LPS-induced rewiring of AM metabolism is critical for memory induction

Tissue-resident AMs predominantly rely on OXPHOS to meet their metabolic demands^37^. This process is tightly linked to the TCA cycle, which serves as the main electron donor for the mitochondrial ETC. Substrates fueling the TCA cycle can be generated by multiple processes, including fatty acid oxidation (FAO), glutaminolysis and oxidation of amino acids or pyruvate^38^. Based on the altered metabolite and lipid composition of LPS-exposed AMs, we speculated that the initial metabolic activation evoked by the training stimulus might be critical for the altered reactivity observed upon secondary challenge. To test this hypothesis, we applied selective metabolic inhibitors during in vitro mexAM training and determined the consequences on the trained IL-6 response exerted upon bacterial challenge. 2-deoxyglucose (2-DG), bis-2-(5-phenylacetamido-1,3,4-thiadiazol-2-yl)ethyl sulfide (BPTES) and etomoxir were used to inhibit glycolysis, glutaminolysis or FAO, respectively (Fig. 5e). While glycolysis appeared to be dispensable for the establishment of mexAM memory, inhibition of FAO and glutaminolysis abrogated the trained IL-6 response of LPS-exposed cells (Fig. 5f), suggesting that LPS-mediated metabolic rewiring is critical for the establishment of AM memory.

### LPS training modulates pneumonia outcome

**Fig. 6:**
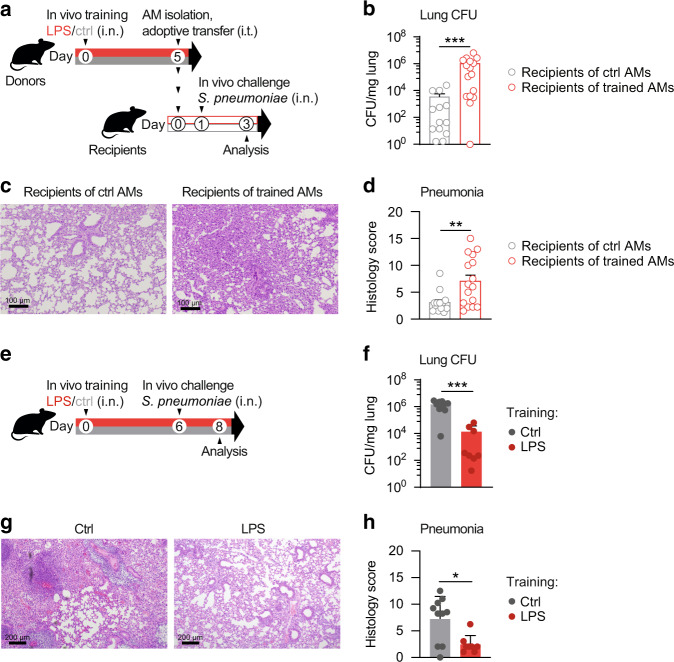
LPS exposure modulates pneumonia outcome in a context-dependent manner. **a** Experimental setup for adoptive transfer of LPS-trained or control donor AMs, followed by i.n. *S. pneumoniae* infection (in vivo challenge). Donor AMs were isolated by BAL five days after in vivo training and transferred intratracheally (i.t.) to naïve WT mice. Recipients were i.n. infected with *S. pneumoniae* 24 h after cell transfer. **b** Lung bacterial loads of recipients, determined 48 h after infection. **c**, **d** Representative histology images (**c**) and pneumonia score (**d**) of H&E-stained lung tissue 48 h after infection; scale bars: 100 µm. Graphs show means + SEM of two pooled experiments with 6–8 biological replicates each (total *n* = 12–16). **e** Experimental setup for in vivo training, followed by infection with *S. pneumoniae* (in vivo challenge) on day six. **f** Lung bacterial loads, 48 h after infection. **g**, **h** Representative histology images (**g**) and pneumonia score (**h**) of H&E-stained lung tissue, 48 h after infection; scale bars: 200 µm. Graphs show means + SD of 8–10 biological replicates. Data are representative of two independent experiments. Statistical analysis: Mann-Whitney-U test. *p ≤ 0.05, **p ≤ 0.01, ***p ≤ 0.001.

AM activation represents an ambiguous balancing act, which serves to promote pathogen clearance while maintaining tissue integrity. Consequently, malfunction, hypo-or hyperactivation of AMs can have detrimental consequences for the host^12,39^. To investigate whether LPS-trained AMs can modulate the outcome of a subsequent pneumococcal infection, we isolated AMs five days after in vivo training (Fig. S7a–c) and transferred them intratracheally (i.t.) into naïve recipients (Fig. S7d), followed by i.n. *S. pneumoniae* infection (*“*in vivo challenge”) 24 h later (Fig. 6a). Recipients of trained AMs displayed increased bacterial loads (Fig. 6b) and lung inflammation (Fig. 6c, d), indicating that adoptive transfer of LPS-experienced AMs impairs host defense against bacterial pneumonia. Considering that LPS-mediated effects on other (i.e. non-AM) cell populations are omitted in a transfer setup, we next addressed the impact of LPS training in a physiologically more relevant setting and infected mice with *S. pneumoniae* six days after LPS exposure (Fig. 6e). These experiments revealed that LPS-pretreated animals displayed enhanced bacterial clearance (Fig. 6f) and reduced lung tissue inflammation (Fig. 6g, h) 48 h after infection. While LPS-exposed animals demonstrated increased recruitment of inflammatory cells early upon infection (6 h; Fig. S7e–f), monocyte and neutrophil numbers were decreased after 48 h (Fig. S7h), indicating accelerated initiation and resolution of inflammation.

Overall, these results highlight the necessity to investigate the physiological consequences of environmental exposures as they may be influenced by multiple cellular players and tissue-specific parameters.

## Discussion

Continuous exposure to environmental microbial triggers poses a strong impact on the education and maturation of our immune system, affecting human health and disease susceptibility^40^. Due to their unique location at the interface of the airways and our environment, AMs are in direct contact with inhaled substances and thus represent potential candidates to develop mucosal-associated trained immunity. Yet, our current knowledge about the tissue-specific properties and consequences of AM memory remains very limited. In this study we discovered that pulmonary exposure to ambient amounts of LPS (corresponding to an inhaled endotoxin concentration of ∼0.5 EU) induces a robust AM memory response, characterized by increased phagocytic activity and cytokine production upon secondary, bacterial challenge. Our RNA-seq and protein analyses collectively revealed that LPS-experienced AMs, being transcriptionally similar to control AMs at baseline, produced elevated amounts of multiple cytokines (e.g. IL-6, IL-12p40 and IL-1β) following subsequent pneumococcal challenge. Given that some of these factors (CXCL1, CXCL2 and CXCL3) are powerful neutrophil chemoattractants that play a prominent role in host defense^41,42^, LPS-induced AM memory may potentially impact the immune response and outcome of infectious challenges by modulating pulmonary inflammation and/or neutrophil recruitment. While AM memory was overall associated with increased production of pro-inflammatory cytokines, we also observed an upregulation of IL-10. This anti-inflammatory mediator was reported to play an ambivalent role in host defense against *S. pneumoniae* as it prevented exacerbated neutrophil influx while favoring bacterial dissemination^43^. In this context, future studies will be required to investigate a potential immunomodulatory effect of trained IL-10 production during pneumococcal infection.

Innate memory responses have mechanistically been linked to epigenetic reprogramming events induced upon exposure to the training stimulus^44^. However, this evidence is primarily based on studies investigating trained immunity in the context of cellular differentiation. For instance, it was reported that systemic administration of Bacille Calmette-Guérin (BCG) promotes bone marrow myelopoiesis by inducing transcriptional changes in hematopoietic stem cells, which give rise to epigenetically modified, trained macrophages^45^. Additionally, β-glucan-trained murine monocytes were shown to differentiate into epigenetically altered macrophages, which confer protection against *Candida albicans* infection^46^. In this study, we characterized the epigenetic profile of AMs upon in vivo training and discovered few changes in chromatin accessibility six days after LPS exposure. In a recent publication, Aegerter et al. demonstrated that tissue-resident AMs displayed minimal changes in chromatin accessibility following an infectious lung insult, whereas monocyte-derived AMs readily maintained an open chromatin conformation^47^. While our data support the notion that AMs exhibit limited epigenetic plasticity, it remains to be investigated whether differential regulation of the identified chromatin loci plays a mechanistic role in the establishment of AM memory. Exploring the possibility that altered baseline deposition of histone marks could mediate AM training, we further demonstrated that selective inhibition of methyl- and acetyltransferase activity did not diminish the training effect. While these findings collectively suggest that chromatin remodeling is no major driving factor of LPS-induced AM memory, it remains to be investigated whether other epigenetic mechanisms (e.g. modulation of gene expression by micro RNAs) or other enzyme classes are critical for AM training.

Despite their limited inflammatory potential at steady state, AMs constitute the front line of cellular host defense against respiratory pathogens and play an important role in the initiation of the inflammatory response^11^. Due to their continuous exchange with the environment, they represent ideal candidates to investigate trained immunity at mucosal sites. Yao et al. demonstrated that respiratory adenoviral infection induces AM memory via CD8^+^ T cell-derived IFN-γ^22^. However, while IFN-γ-exposed AMs showed enhanced responsiveness upon subsequent *S. pneumoniae* challenge, the cells did not return to a baseline state following adenoviral infection, which was illustrated by elevated glycolysis and increased transcriptional activity 28 days after adenoviral exposure. These findings imply that the increased reactivity of memory AMs upon secondary challenge may possibly be a consequence of prior IFN-γ-priming, rather than innate training. While IFN-γ-priming constitutes a well-established concept and reportedly alters macrophage immunity by promoting a proinflammatory phenotype^48–50^, we showed that LPS-induced AM training occurs *independently* of IFN-γ-receptor signaling and adaptive immunity, and, instead, identified a novel mechanistic regulation of pulmonary macrophage memory. We discovered that type1 IFN deficiency profoundly diminishes AM training and extended our findings by showing that i.n. administration of IFN-β can replicate the training effect of pulmonary LPS exposure. While type 1 IFNs possess potent antiviral and immunostimulatory properties, mistimed, inappropriate or excessive type 1 IFN responses can impair anti-bacterial immunity, and thus facilitate secondary bacterial superinfections, e.g. via suppression of Th17 responses^51,52^, or impairment of neutrophil recruitment^53^. Notably, our experimental model differs from settings of viral-bacterial superinfections in important aspects, such as the extent and duration of type 1 IFN exposure, as well as the timing of *S. pneumoniae* infection. As we have not investigated a potential impact of i.n. IFN-β treatment on pneumonia outcome in this study, future research will be required to assess potential consequences of exogenous IFN-β administration on pneumococcal clearance.

Recent research has highlighted the critical impact of cellular metabolism on immune cell activation and regulation of innate memory responses^32^. Along these lines, multiple studies provide evidence for a key role of glycolytic metabolism in trained monocytes^31^. In addition to glycolysis, glutaminolysis has been reported to be essential for the induction of BCG-induced innate memory^54^ and both pathways have been shown to be closely intertwined with epigenetic regulation. While these studies have highlighted a critical role of metabolic regulation in trained immunity in vitro, we lack knowledge about tissue-specific metabolic reprogramming of innate immune cells.

The pulmonary niche constitutes a unique mucosal environment, which is characterized by remarkably low glucose availability^55^. Being metabolically adapted to these conditions, AMs display limited glycolytic activity and rely on mitochondrial  OXPHOS^14,37^. A recent study by Svedberg et al. demonstrated that impaired glycolysis of AMs limits their responsiveness during type 2 inflammation^56^, suggesting a functional implication of this metabolic constraint. We here identified a critical role of glutaminolysis and FAO in the establishment of LPS-induced AM memory and provide evidence that glycolytic activation is dispensable for this process. Of note, the altered metabolic profile of AMs was not reflected on a gene expression level, which might potentially be explained by different temporal dynamics of metabolic and genetic responses. To our knowledge, this study is the first to describe a metabolic dependency of AM memory, and to report a role of FAO in trained immunity of tissue-resident macrophages. Furthermore, these data emphasize that the cellular characteristics of AM memory reflect their unique immunological and metabolic properties.

In recent years, several epidemiological and experimental studies have identified a beneficial role of trained immunity due to heterologous protection against unrelated pathogens^46,57,58^. However, innate memory responses may also be maladaptive in conditions of chronic inflammation, such as atherosclerosis, neurodegeneration and autoimmunity^59^. In this study, we found that adoptive transfer of trained AMs resulted in increased lung inflammation and impaired bacterial clearance following *S. pneumoniae* infection (compared to recipients of control AMs). In contrast, i.n. exposure to LPS, a scenario in which LPS-mediated reprogramming can potentially affect any lung-resident cell population, improved pneumonia outcome. Based on these findings, and disregarding the inherent limitations of cell transfer experiments due to concomitant inflammation, we speculate that i.n. LPS treatment not only induces AM training but likely imprints other (non-AM) cell populations (e.g. resident structural, myeloid or lymphoid cell types), thereby altering their responsiveness to *S. pneumoniae* challenge. Such additional reprogramming events might be crucial to prevent excessive inflammation and promote anti-bacterial immunity. Similarly, LPS-induced modulations of the local immune and metabolic environment (e.g. altered availability of soluble mediators or metabolites) may critically influence the outcome of subsequent infectious challenges. While our data indicate that trained AMs can exert a significant impact on pneumococcal clearance in a transfer setting, the experimental set up did not enable us to delineate whether and how AMs contribute to the protective effect following intranasal LPS exposure. These findings may therefore represent two independent observations. However, they collectively underline the complexity of biological systems and illustrate that a single cell population may not be sufficient to dictate the ultimate outcome of host defense.

Several epidemiological studies have demonstrated that exposure to a microbe-rich, diverse environment is linked to a decreased prevalence of allergies, a phenomenon commonly referred to as the “farm effect”^10^. A remarkable example for this effect is offered by a study showing that house dust from traditional farming environment decreases asthma prevalence and development by engaging and modulating innate immunity^60^. We here demonstrated that inhalation-exposure to ambient endotoxin levels significantly improves the outcome of bacterial pneumonia. While these findings underline the immunomodulatory potential of environmental agents, future studies will be required to dissect the underlying cellular mechanisms of this observation. Furthermore, it remains to be investigated whether LPS-induced reprogramming of AMs plays a role in allergic airway inflammation and other respiratory diseases.

Altogether, our data highlight the necessity to investigate trained immunity in a tissue-specific context and emphasize that the mechanisms and consequences of innate memory are influenced by the local microenvironment and disease setting.

## Materials and methods

Additional methodological details on cell isolation and flow cytometry, phagocytosis/efferocytosis assays, RNA-seq, ATAC-seq and LC-MS/MS sample preparation and data analysis, mexAM culture, histological evaluation and study design can be found in the Supplementary Materials.

### Mice

Age-matched, 8–10-week-old male mice were used throughout the study. Mice were housed at the Medical University of Vienna (MUW, Austria), at the Institute of Molecular Biotechnology (IMBA, Vienna, Austria), at the Vienna BioCenter (VBC, Austria) or at the Max Planck Institute (MPI) of Immunobiology and Epigenetics (Freiburg, Germany). C57BL/6 J mice were purchased from Janvier (in-house maintenance breeding at MUW) or from the Jackson Laboratory (maintenance at MPI). Rag2^−/−^ mice^61^ (originally ordered from Jackson), Ifnar1^ΔCD169^ and Ifnar1^fl/fl^ control mice were bred at IMBA. Ifnar1^−/−^^62^, Ifngr1^−/−^^63^, and respective C57BL/6 N wild type control mice were bred at the VBC. All mice were housed in a specific pathogen-free environment according to the Federation of European Laboratory Animal Science Associations (FELASA) guidelines and were matched for sex, age and genetic background in individual experiments. All mouse experiments were approved by and performed in accordance with the Austrian Federal Ministry of Science and Research (BMWF-66.009/0363-WF/V/3b/2017; 2020-0.009.488) and the Regierungspraesidium Freiburg, Germany (35-9185.81/G-18/65).

### In vivo training

Mice received 50 µL endotoxin-free saline (Braun) containing 1 ng LPS (Sigma; *E.coli* O55:B5) or 2000 U mouse IFN-β (pbl assay science) or saline only i.n. under light isoflurane anaesthesia (2% isoflurane, 2 L/min O_2_) or after intraperitoneal (i.p.) injection of Ketasol (100 mg/kg; OGRIS Pharma) and Rompun (10 mg/kg; Bayer).

### Ex vivo challenge of AMs

On day six after in vivo training, AMs were isolated by BAL as described (s. Supplementary Materials). Cells were resuspended in RPMI medium (10% FCS, 1% penicillin-streptomycin [PS; Sigma]), counted and seeded at a density of 5 × 10^4^ cells per well in a TC-treated 96 well plate (Corning). After 2 h, non-adherent cells were washed off with PBS. Subsequently, trained and control AMs were challenged with heat-inactivated *S. pneumoniae* (HISP; ATCC6303, MOI [multiplicity of infection] 100) in RPMI medium (3% FCS, 1% PS) or with medium only for 3 h (RNA-seq analysis) or 16–24 h (cytokine analysis, Seahorse experiments). IL-6 levels were quantified by ELISA (BioLegend) or LEGENDplex (BioLegend). Levels of CXCL1, TGF-β1, G-CSF, IL-18, IL-23, CCL22, IL-10, IL-12p40, IL-12p70, IL-6, CCL17 and IL-1β were measured using the LEGENDplex Mouse Macrophage/Microglia Panel (BioLegend) according to the manufacturer’s instructions. Data analysis was performed using the LEGENDplex data analysis software.

### In vivo labeling of tissue-resident AMs

Tissue-resident AMs were labeled by i.n. treatment with PKH26 (Sigma) eight days prior to in vivo training. The dye was prepared according to the manufacturer’s instructions and administered at 10 µM in a volume of 50 µL. At indicated time points, BALF and post-lavage lung AMs were analyzed by flow cytometry to determine frequencies of PKH26^+^ and PKH26^-^ cells.

### Seahorse analysis

AMs were isolated by BAL six days after in vivo training. Biological replicates were pooled by experimental group and seeded as technical replicates in XF-96 cell culture plates (Agilent) at a density of 8 × 10^4^ cells/well in 80 µL RPMI medium (3% FCS, 1% PS). To remove non-adherent cells, the plate was incubated for 2 h at 37 °C and cells were washed twice either with PBS (followed by subsequent ex vivo challenge, performed as described) or XF assay medium (Seahorse XF RPMI medium, pH 7.4, 10 mM Glucose, 1 mM Pyruvate, 2mM L-Glutamine [all from Agilent], 3% FCS). Prior to analysis, cells were incubated under non-CO_2_ conditions in XF assay medium for 1 h. Oxygen consumption rate (OCR) and extracellular acidification rate (ECAR) of AMs were analyzed using a Seahorse XF-96 Extracellular Flux Analyzer (Agilent). Where indicated, 1 μM oligomycin, 1.5 μM carbonyl cyanide p-trifluoromethoxyphenylhydrazone (FCCP) or 100 nM rotenone plus 1 μM antimycin A (R/A; all from Sigma) were injected to assess mitochondrial function. Means of R/A values (non-mitochondrial respiration) were subtracted from OCR raw data for quantification of mitochondrial parameters. ECAR data represent raw values.

### Murine pneumonia model

Pneumonia was induced by i.n. infection with 10^4^ CFUs mid-logarithmic-stage *S. pneumoniae* serotype 3 (ATCC6303) as previously described^64^. Lungs were harvested 48 h after infection. Right lobes were collected for determination of bacterial counts, the left lobe was collected for histological analysis. Bacterial growth was quantified by plating 10-fold serial dilutions of lung homogenates on blood agar plates.

### Adoptive AM transfer

For adoptive AM transfer experiments, donor AMs were isolated by BAL on day five after in vivo training with LPS or saline. BALF samples were pooled by experimental group and centrifuged for 5 min (4 °C, 300 g). Cells were resuspended in PBS, counted and diluted to a concentration of 10^7^ cells/ mL. Subsequently, 3 × 10^5^ cells were transferred intratracheally to naïve wild-type recipients in a volume of 30 µL. Twenty-four hours after transfer (i.e. six days after donor training), recipients were i.n. infected with 10^4^ CFU *S. pneumoniae*.

### Statistical analysis

Differences in values obtained from two experimental groups were assessed by student’s *t*-test (unpaired) or Mann-Whitney test (non-parametric). One-way ANOVA or two-way ANOVA analysis followed by Šídák’s multiple comparisons test was used to determine differences between multiple groups. Data were analyzed using GraphPad Prism 8.0 and are presented as mean + SD or mean + SEM for experiments performed with biological or technical replicates, respectively.

## Supplementary information

Supplemental Material & Methods

author checklist

Supplementary Table S1

Supplementary Table S2

Supplementary Table S3

Supplementary Table S4

## Data Availability

Raw and processed sequencing data (RNA-seq and ATAC-seq) are available in the NCBI Gene Expression Omnibus database (accession number GSE184684). Raw and processed LC-MS/MS data are available in the MetaboLights database (accession number MTBLS3151).
