# Supplementary material for: Trained immunity of alveolar macrophages requires metabolic rewiring and type 1 interferon signaling
Source: Mucosal Immunol. 2022 Jul 18;15(5):896–907. doi: 10.1038/s41385-022-00528-5 (PMC9385480; doi:10.1038/s41385-022-00528-5)
Supplement: Supplementary file 1 — Supplemental Material & Methods [file 41385_2022_528_MOESM1_ESM.pdf]

Supplementary Materials & Methods:

Cell isolation and flow cytometry (BAL, lung)

BAL fluid was collected as previously described with minor modifications[1]. In brief, after exposure and midline incision of the trachea, mouse airways were flushed with a total of 5 mL endotoxin-free saline by instillation of 0.5 mL aliquots via a sterile 18-gauge tracheal cannula (Venflon, BD). Prior to staining, BALF samples were pelleted by centrifugation (4°C, 300g) and resuspended in PBS 1% BSA. Post-lavage lungs were weighed, and tissue homogenization was performed using a gentleMACS™ Tissue Dissociator (Miltenyi Biotec) following the manufacturer's protocol with minor adaptations. Briefly, after primary homogenization (program m_lung_01_02), lungs were digested for 35 min on a shaker (37°C, 180 rpm) in RPMI medium supplemented with 5% FCS (Sigma), DNase I (12 U/ mL, Sigma) and Collagenase I (160U/ mL, Gibco). Subsequently, suspensions were homogenized (program m_lung_02_01), filtered over 70 µM strainers (Miltenyi Biotec) and centrifuged for 5 min (4°C, 300 g). Erythrocytes were lysed on ice in ACK lysis buffer (150 mM NH_4Cl, 10 mM $KHCO_3$, 0.1 mM Na_2EDTA, pH 7.2 – 7.4; all chemicals from Sigma), cell suspensions were filtered over 30 µM strainers and resuspended in PBS 1% BSA. BALF and lung cell suspensions were incubated with viability dye (eBioscience) and anti-mouse CD16/32 (eBioscience), followed by fluorescently labeled monoclonal antibodies (Table S4). Stained cells were washed with PBS and fixed for 30 min using Fix&Perm Fixation Medium A (Nordic Mubio) unless indicated otherwise. Samples were acquired on an LSR Fortessa flow cytometer (BD) and analyzed using FlowJo software (FlowJo LLC). For LC-MS/MS analysis, $CD11c^+$ Siglec F^+ AMs were flow-sorted on a FACSAria™ Fusion flow cytometer (BD).

Phagocytosis assay

AM phagocytosis was assessed on day six after *in vivo* training with LPS or saline. AMs were isolated by BAL, seeded at equal numbers and left to adhere in RPMI medium (10% FCS, 1% PS). After 2 h, cells were washed with PBS and incubated with FITC-labeled HISP (MOI 100) in RPMI medium (3% FCS, 1% PS) at 37°C or 4°C (negative control) for 45 min. Thereafter, cells were washed again and treated with proteinase K (50 µg/mL; Roche) for 10 min at 4°C to remove residual adherent bacteria. After washing, samples were processed for flow cytometry as described and incubated with fluorescently labeled monoclonal antibodies (anti-CD45, anti-CD11c and anti-Siglec F; Table S4) to allow identification of AMs. Cells were analyzed by flow cytometry without prior fixation. The phagocytosis index was calculated as [(37°C %$FITC^+$ AMs)*(37°C FITC MFI of $FITC^+$ AMs)]-[(4°C %$FITC^+$ AMs)*(4°C FITC MFI of $FITC^+$ AMs)].

Adoptive transfer of CFSE-labeled apoptotic thymocytes (efferocytosis assay)

For efferocytosis assays, thymocytes were isolated from 4-week old wild type mice, seeded at a density of 1.8×10^7 cells/well in a non-coated 12-well plate (CytoOne) and incubated in RPMI medium (10% FCS, 1% PS) containing dexamethasone (1 µM, Sigma). After 24 h, the apoptotic cells were collected by centrifugation (2500 rpm, 7 min, 4°C), washed twice with PBS, diluted to a concentration of 1×10^7 cells/mL and incubated with CFSE (5 µM, Invitrogen) for 20 min at RT. Subsequently, labeled cells were washed twice and resuspended at a concentration of 1×10^8 cells/mL in PBS. WT mice received 3×10^6 CFSE-labeled apoptotic thymocytes in 30 µL PBS (or PBS only) intratracheally (i.t.) on day six after i.n. LPS/saline treatment. Two hours after transfer, AMs were isolated by BAL, processed for flow cytometry as described and incubated with fluorescently labeled monoclonal antibodies (anti-CD45, anti-CD11c, anti-Siglec F, anti-MerTK, anti-Axl and Ter119; Table S4). Cells were analyzed by flow cytometry without prior fixation.

RNA isolation & Quant-seq analysis

RNA-seq analysis was performed on day six after *in vivo* training. AMs were isolated and challenged as described. Three hours after *ex vivo* challenge, cells were washed twice with cold PBS and lysed in RLT buffer (Qiagen) containing 1% 2-mercaptoethanol (Sigma). Total RNA was isolated using the RNeasy Micro kit (Qiagen). QuantSeq libraries were prepared using the QuantSeq 3' mRNA-Seq Library Prep Kit (FWD) for Illumina in combination with the PCR Add-on Kit for Illumina and the UMI Second Strand Synthesis Module for QuantSeq FWD (all from Lexogen), following the manufacturers' instructions. Libraries were prepared with 150 ng total RNA input and 15 amplification cycles. Sample quality was assessed on a Bioanalyzer 2100 (Agilent Technologies). Subsequently, 65 bp single-end sequencing was performed by the Biomedical Sequencing Facility (BSF, Research Center for Molecular Medicine and Medical University of Vienna) on a HiSeq3000/4000 instrument (Illumina).

Quant-seq data processing and bioinformatic analysis

Raw sequencing data were processed using the QuantSeq data analysis pipeline (Lexogen) hosted on the BlueBee Genomics Platform (Bluebee), as recommended by the manufacturer. In brief, unique molecular identifier (UMIs) were added to the read identifiers and trimmed from the reads using the *umi2index* process. Next, reads were quality- and adapter-trimmed using *Bbduk* and aligned to the mouse reference genome (mm10) using STAR aligner with modified ENCODE settings. To remove PCR duplicates, reads with identical UMIs and mapping were collapsed. Finally, reads mapping to genes were quantified using *HTSeq-count*. Differential gene expression was assessed using *DESeq2*[2]. Results were corrected for multiple testing using independent hypothesis weighting (*ihw* R package)[3]. Genes with an FDR-adjusted p-value of ≤ 0.1 were considered differentially expressed. Differentially activated KEGG pathways were assessed using Signaling Pathway Impact Analysis (SPIA)[4].

Assay for transposase-accessible chromatin with sequencing (ATAC-seq)

To analyze chromatin accessibility, AMs were isolated six days after *in vivo* training by BAL. Differentially accessible regions of biological replicates were identified by ATAC-seq as previously described with some adaptations[5, 6]. Briefly, $5x10^4$ AMs were pelleted by centrifugation (4°C, 5 min, 500 g). Each pellet was lysed in 25 µL transposase reaction mix (9.75 µL RNase-free water, 12.5 µL 2 x TD buffer [Illumina], 0.5 µL 50x proteinase inhibitor cocktail [Roche], 2 µL TDE1 [Illumina], 0.25 µL 1% Digitonin [Promega]) and incubated at 37°C for 30 min. Subsequently, DNA was purified using the Qiagen MinElute kit and eluted in 12 µL. The optimal number of amplification cycles was determined for each sample by qPCR (qPCR reaction mix per sample: 2.7 µL RNAse-free water, 0.2 µL ROX reference dye [Invitrogen], 0.5 µL index primer 1 noMX, 0.5 µL index primer 2.1, 0.1 µL 100x SYBR green [Sigma-Aldrich], 5 µL NEBnext High-Fidelity 2x PCR master mix [New England Biolabs] and 1 µL tagmented sample). Subsequent library amplification was performed at the determined cycle numbers, using custom Nextera index primers[6] (enrichment PCR reaction mix per sample: 10 µL RNAse-free water, 2.5 µL index primer 1 noMX, 2.5 µL barcoded index primer, 25 µL NEBnext High-Fidelity 2x PCR master mix, 10 µL tagmented sample). Enrichment was followed by SPRI (Beckman Coulter) size selection in order to exclude DNA fragments exceeding 1200 bp. DNA concentrations were determined using a Qubit fluorometer (Life Technologies). Libraries were pooled at a final concentration of 4 nM and sequenced by the BSF (HiSeq 3000/4000, 50 bp single-end).

ATAC-seq data processing and bioinformatic analysis

Quality of raw fastq files was assessed using fastqc (v.0.11.8). Subsequently, raw reads were trimmed with trimmomatic (v.0.32) and aligned to the mouse reference genome (mm10) using bowtie (v.2.2.4; parameters: very sensitive, end-to-end). For further analysis, primary alignments with a mapping quality below 30 were discarded. Peak-calling was performed using MACS (v.2.1.0; parameters: nomodel, shift -100, extsize 200). Peak files were loaded into R (v.4.1.0) and filtered to only retain peaks, which were confirmed by at least three samples within each treatment group (LPS or control). Next, a consensus peak set was computed by applying the function *reduce* of the GenomicRanges (v.1.42.0) package. Consensus peaks overlapping with blacklisted genomic regions were discarded (source: http://mitra.stanford.edu/kundaje/akundaje/release/blacklists/mm10-mouse/).

Quantitative measurements were obtained by counting reads within consensus peaks using the function *featureCounts* of the Rsubread (v.2.4.3) package in R. The peak count matrix was filtered using the edgeR (v.3.32.1) function *filterByExpr* and peaks with a minimum of 100 counts in at least 75% of the samples of a treatment group were retained. Sample-specific quality weights were computed with the *voomWithQualityWeights* function of the limma (3.46.0) R package using the trimmed mean of M values (TMM). For each peak region, a linear model was fitted to the count data with lmFit and DARs were determined with eBayes. DARs with an adjusted p-value ≤ 0.05 were

considered significant. Finally, peak regions were annotated to the org.Mm.eg.db reference using the ChIPseeker (v.1.5.1) function *annotatePeak*.

LC-MS/MS sample prep and analysis

For metabolomic and lipidomic analyses of trained and control AMs, $CD11c^+$ Siglec F^+ AMs were flow-sorted from BALFs six days after *in vivo* administration of LPS or saline (biological replicates). Each cell pellet was lysed in 310 µL MetOH. After adding 10 µL isotopically-labelled lipid internal standard mix (dissolved in MetOH) and 80 µL isotopically-labelled metabolite internal standard mix (dissolved in ddH_2O), samples were vortexed and transferred to 1.5 mL HPLC glass vials. Subsequently, 640 µL chloroform were added and samples were vortexed again. After addition of 240 µL ddH_2O, samples were shaken on ice (10 min, 400 rpm) and centrifuged (4°C, 10 min, 1000 g). The upper water phase was collected and dried using a nitrogen evaporator. Samples were reconstituted in 50 µL ddH_2O for LC-MS based metabolite measurements. The lower chloroform phase was collected and dried using speed vac. Samples were reconstituted in 20 µL MetOH for LC-MS based lipid measurements. LC-MS metabolite analysis (detection of amino acids) was performed using a Vanquish UHPLC system coupled with an Orbitrap Q Exactive (Thermo Scientific) mass spectrometer. LC-MS/MS analysis (detection of TCA cycle metabolites) was performed on a Xevo TQ-MS (Waters) mass spectrometer using an Acquity UHPLC (Waters) system. LC-MS lipid analysis was performed using a Vanquish UHPLC system (Thermo Fisher Scientific) combined with an Orbitrap Fusion Lumos Tribrid mass spectrometer (Thermo Fisher Scientific). Data were processed using the TraceFinder software (ThermoFisher Scientific; Analysis of amino acids and lipids) and the MassLynx V4.1 software (Waters; Analysis of TCA cycle metabolites).

LC-MS/MS data processing and bioinformatic analysis

Bioinformatic analyses were performed in R (v. 3.4.1). For metabolite data analysis, individual metabolite concentrations were normalized to the total number of sorted cells. Differences between groups were assessed using t-tests. Individual metabolites with p-values ≤ 0.05 and individual amino acids with p-values ≤ 0.05 and FDR ≤ 0.2 were considered statistically significant. For lipid data analysis, individual lipid species that were below detection limit in more than 66% of all samples were excluded from further analysis. For the remaining lipids, values below detection limit were imputed using the half minimum method. To account for differences in sorted cell numbers and potential variation induced by sample processing, lipid data were normalized by centered log-ratio transformation. Lipids significantly differing between groups were identified using t-tests, and individual lipid species with p-values ≤ 0.05 and FDR ≤ 0.2 were considered statistically significant.

Generation and *in vitro* training of murine *ex vivo* cultured alveolar macrophages (mexAMs)

For generation of mexAM cultures, BAL AMs were obtained from adult wild type mice, and expanded and cultured in mexAM medium (RPMI supplemented with 10% FCS, 1% PS, 30 ng/ mL murine GM-CSF [Peprotech], 10 ng/ mL human TGF-ß [Peprotech] and 1 µM rosiglitazone [Sigma]) as previously described[7]. For *in vitro* training, mexAMs (passage 15-25) were seeded in 6-well plates (Corning; $4x10^5$ cells/well in 2 mL mexAM medium) and stimulated with 10 ng/ mL LPS (Sigma) or 400 U/ mL mouse IFN-β (pbl assay science) for 24 h. To address the role of metabolic and epigenetic regulation, cells were pre-incubated for 1 h in mexAM medium containing 1 mM 2-deoxyglucose (2-DG; Sigma), 10 µM bis-2-(5-phenylacetamido-1,3,4-thiadiazol-2-yl)ethyl sulfide (BPTES; Sigma), 3 µM etomoxir (Sigma), 1 mM 5'-deoxy-5'-methylthioadenosine (MTA; Cayman chemical), 10 µM anacardic acid (Abcam) or DMSO, followed by incubation with LPS or medium in presence of inhibitors or DMSO for 24 h. After stimulation, cells were washed with PBS and maintained in mexAM medium. On day three, 0.5 mL fresh medium were added on top. On day six, cells were washed with PBS, detached by Lidocaine (Xylanaest purum; Gebro Pharma; 0.5% in PBS) treatment and seeded in a 96 well plate (Corning) in RPMI medium (3% FCS, 1% PS; $5x10^4$ cells/well). After 2 h of adherence, cells were challenged with HISP (MOI 100 in RPMI medium) or medium only for 16 h.

Histological analysis

For histological analysis, lungs were fixed in 10% formaldehyde and embedded in paraffin. Lung sections were H&E-stained and examined by a trained pathologist (FO), blinded to experimental group assignments. Examined parameters included pleuritis, edema, bronchitis, endothelitis, interstitial inflammation and perivascular infiltrates. Each parameter was scored in the range of 0 to 3 points (0=absent, 1=mild, 2=moderate, 3=severe). The final histology score was calculated as the sum of all scores for the indicated parameters with additional 0.5 points added for every infiltrate covering 10% of total tissue area.

Study design

The aim of this study was to investigate whether environmental LPS exposure alters AM reactivity to a subsequent bacterial challenge by inducing trained immunity. To this end, we i.n. administered LPS or saline (control) to male WT mice. LPS-mediated effects on secondary AM responses were assessed by ELISA/LEGENDplex and FACS analysis at baseline and upon *ex vivo* HISP challenge (day six after treatment). Flow cytometry-based *in vivo* labeling of resident AMs was conducted to evaluate a potential contribution of recruited monocytes to the trained AM pool. We investigated the role of adaptive immunity, interferon-α/β-receptor- and IFN-γ-receptor signaling in LPS-induced AM memory by applying our training model (*in vivo* training, followed by *ex vivo* AM challenge) to $Rag2^{-/-}$, $Ifnar1^{-/-}$, $Ifnar1^{\Delta CD169}$, $Ifngr1^{-/-}$ and respective control mice. Furthermore, we i.n. administered recombinant IFN-β and analyzed AM IL-6 production upon *ex vivo* HISP challenge. In order to

characterize the genetic, epigenetic and metabolic profile of LPS-trained AMs, we performed RNA-seq, ATAC-seq, metabolomic/lipidomic and Seahorse analyses. The role of epigenetic regulation and metabolism was further evaluated using selective epigenetic or metabolic inhibitors. To assess a potential impact of trained AMs on pneumonia outcome, we performed an adoptive intratracheal transfer of trained or control AMs into naïve recipients, which were subsequently infected with *S. pneumoniae*. Lastly, to investigate the physiological consequence of LPS inhalation, we i.n. administered LPS or saline (control) to wild type mice, followed by *S. pneumoniae* infection six days later. Throughout the study, mice were randomly distributed to experimental cages prior to treatment to avoid potential cage effects. To minimize confounding effects of animal housing/location, imported strains were maintained in the in-house mouse facility for a minimum of two weeks. For pneumonia experiments, termination criteria (BMWF-66.009/0363-WF/V/3b/2017; 2020-0.009.488) were established a priori. For remaining experiments, no a priori inclusion/exclusion criteria were set. Histological samples were evaluated by a trained pathologist (FO), blinded to experimental group assignments.

Supplementary References:

1. Rijneveld, A.W. *et al.* TNF-alpha compensates for the impaired host defense of IL-1 type I receptor-deficient mice during pneumococcal pneumonia. *J Immunol* **167**, 5240-5246 (2001).

2. Love, M.I., Huber, W. & Anders, S. Moderated estimation of fold change and dispersion for RNA-seq data with DESeq2. *Genome Biol* **15**, 550 (2014).

3. Ignatiadis, N., Klaus, B., Zaugg, J.B. & Huber, W. Data-driven hypothesis weighting increases detection power in genome-scale multiple testing. *Nat Methods* **13**, 577-580 (2016).

4. Tarca, A.L. *et al.* A novel signaling pathway impact analysis. *Bioinformatics* **25**, 75-82 (2009).

5. Corces, M.R. *et al.* Lineage-specific and single-cell chromatin accessibility charts human hematopoiesis and leukemia evolution. *Nat Genet* **48**, 1193-1203 (2016).

6. Buenrostro, J.D., Giresi, P.G., Zaba, L.C., Chang, H.Y. & Greenleaf, W.J. Transposition of native chromatin for fast and sensitive epigenomic profiling of open chromatin, DNA-binding proteins and nucleosome position. *Nat Methods* **10**, 1213-1218 (2013).

7. Gorki, A.D. *et al.* Murine Ex Vivo Cultured Alveolar Macrophages Provide a Novel Tool to Study Tissue-Resident Macrophage Behavior and Function. *Am J Respir Cell Mol Biol* **66**, 64-75 (2022).

Fig. S1

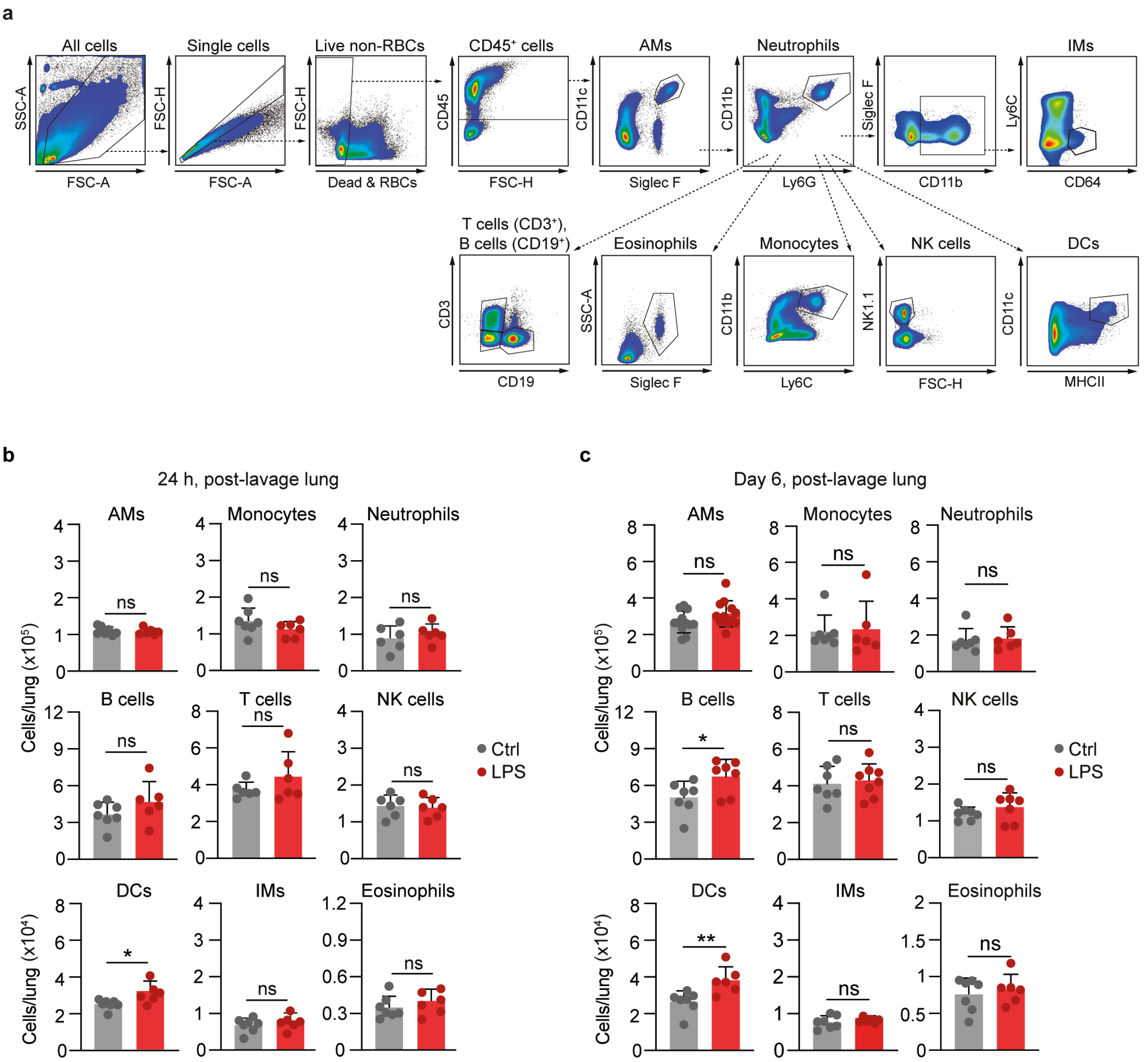

Fig. S1. FACS analysis of post-lavage lung tissue following LPS exposure.

Refers to Fig. 1. **(a)** Flow cytometry gating strategy for analysis of post-lavage lungs following i.n. LPS (1 ng/mouse) or saline exposure. **(b, c)** Cellular composition of post-lavage lungs, 24 h **(b)** and six days **(c)** after treatment. Graphs show means + SD of 6-12 biological replicates. Data are representative of two independent experiments. Statistical analysis: student's t-test. ns, not significant. *p ≤ 0.05, **p ≤ 0.01.

Fig. S2

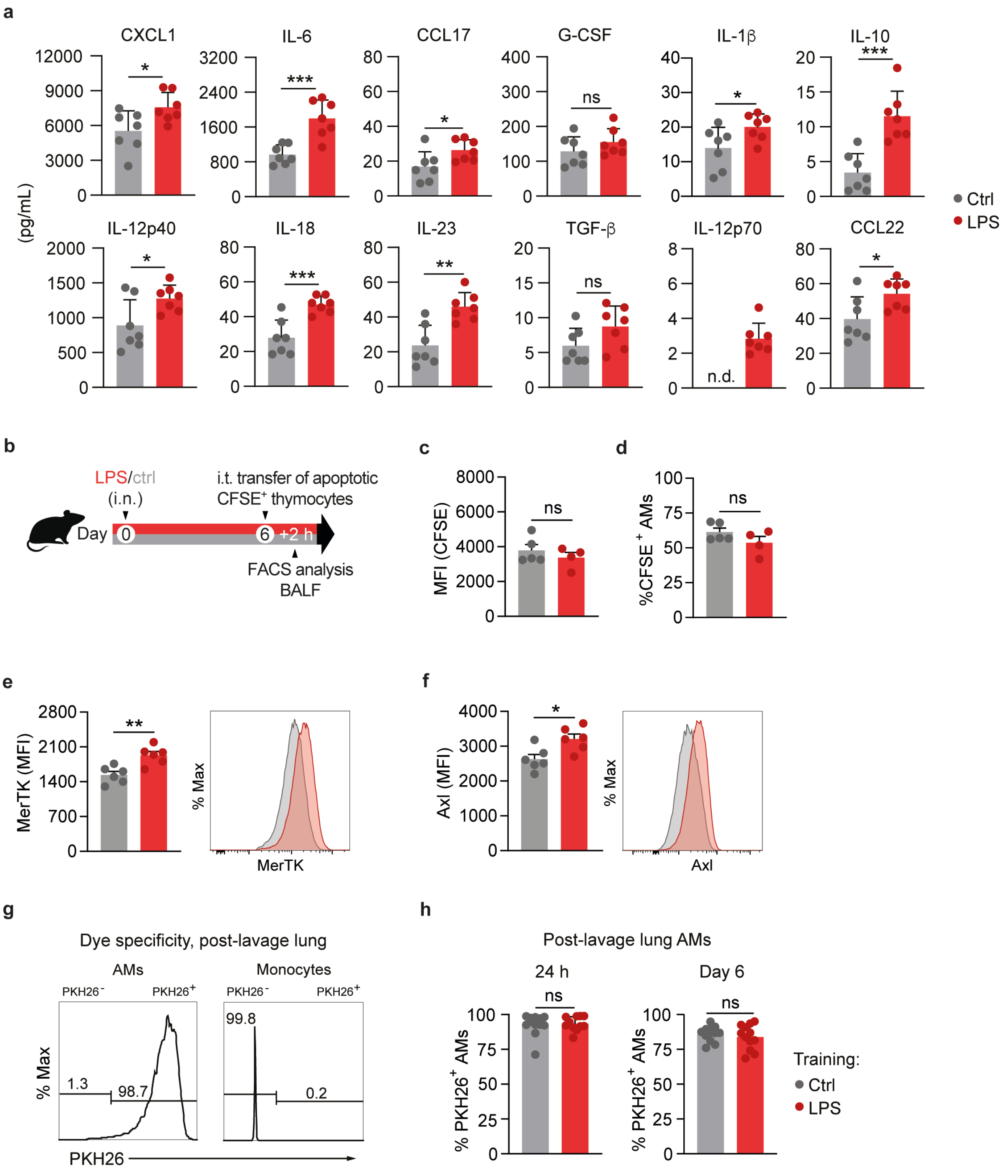
a
CXCL1
IL-6
CCL17
G-CSF
IL-1β
IL-10
IL-12p40
IL-18
IL-23
TGF-β
IL-12p70
CCL22
(pg/mL)
n.d.
ns
Ctrl
LPS
b
LPS/ctrl
(i.n.)
i.t. transfer of apoptotic
CFSE+ thymocytes
Day 0
6 +2 h
FACS analysis
BALF
c
MFI (CFSE)
d
%CFSE+ AMs
e
MerTK (MFI)
% Max
MerTK
f
Axl (MFI)
% Max
Axl
g
Dye specificity, post-lavage lung
AMs
Monocytes
PKH26-
PKH26+
% Max
1.3
98.7
99.8
0.2
PKH26
h
Post-lavage lung AMs
24 h
Day 6
% PKH26+ AMs
Training:
Ctrl
LPS

Fig. S2. Analysis of AM cytokines, efferocytosis and turnover following *in vivo* LPS exposure.

Refers to Fig. 1. **(a)** Absolute cytokine levels of LPS-exposed and control AMs upon *ex vivo* HISP challenge (16 h), determined by LEGENDplex analysis. BAL AMs were isolated six days after *in vivo* treatment. **(b)** Experimental setup for analysis of AM efferocytosis. Six days after *in vivo* LPS/saline exposure, WT mice received $3x10^6$ CFSE-labeled apoptotic thymocytes via intratracheal transfer. BALF was collected and subjected to FACS analysis 2 h after transfer. **(c, d)** CFSE mean fluorescence intensity (MFI) **(c)** and percentage of $CFSE^+$ cells **(d)**, pre-gated on $CD11c^+$ Siglec F^+ BALF AMs. **(e, f)** MerTK **(e)** and Axl **(f)** MFI of $CD11c^+$ Siglec F^+ BALF AMs following apoptotic thymocyte transfer. Representative histograms are displayed adjacent to bar graphs. Data are representative of two independent experiments. **(g)** Representative histograms depicting PKH26 MFI of $CD11c^+$ Siglec F^+ post-lavage lung AMs and $CD11b^+$ $Ly6C^+$ monocytes, 24 h after *in vivo* training. **(h)** Percentage of $PKH26^+$ post-lavage lung AMs 24 h and six days after training. Graphs show means + SD of 7-8 **(a)**, 4-6 **(c-f)** or 11-12 **(h)** biological replicates. Statistical analysis: student's t-test. ns, not significant. $*p \leq 0.05$, $**p \leq 0.01$, $***p \leq 0.001$.

Fig. S3

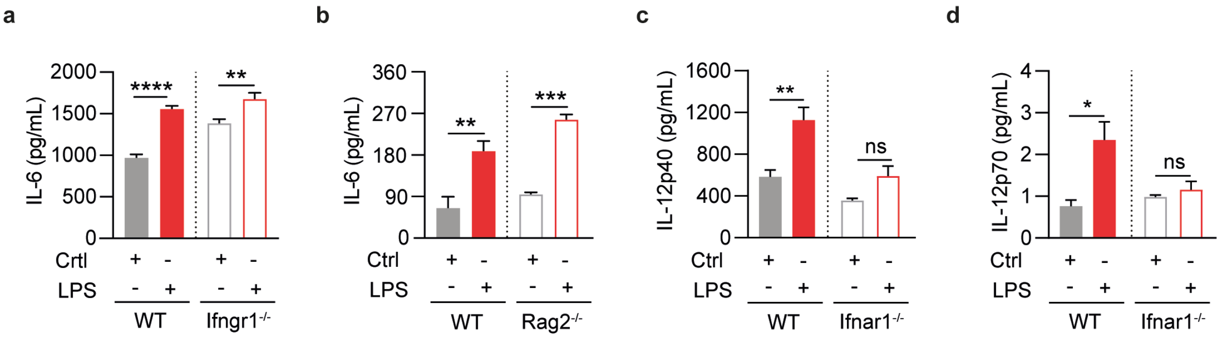

Fig. S3. Cytokine analysis of HISP-challenged Ifngr1$^{-/-}$, Rag2$^{-/-}$ and Ifnar1$^{-/-}$ AMs six days after *in vivo* training.

Refers to Fig. 2. **(a, b)** IL-6 levels of HISP-challenged (16 h) LPS-exposed and control AMs, isolated six days after *in vivo* training of Ifngr1$^{-/-}$ **(a)**, Rag2$^{-/-}$ **(b)** and respective wild type control mice. **(c, d)** IL-12p40 **(c)** and IL-12p70 **(d)** levels of HISP-challenged LPS-exposed and control AMs, isolated six days after *in vivo* training of Ifnar1$^{-/-}$ and WT control mice. Biological replicates (n=4) were pooled and seeded as technical replicates. Graphs represent means + SEM of 3-5 technical replicates. Data are representative of two independent experiments. Statistical analysis: 2-way ANOVA (factor 1: training; factor 2: genotype). ns, not significant. *p ≤ 0.05, **p ≤ 0.01, ***p ≤ 0.001, ****p ≤ 0.0001.

Fig. S4

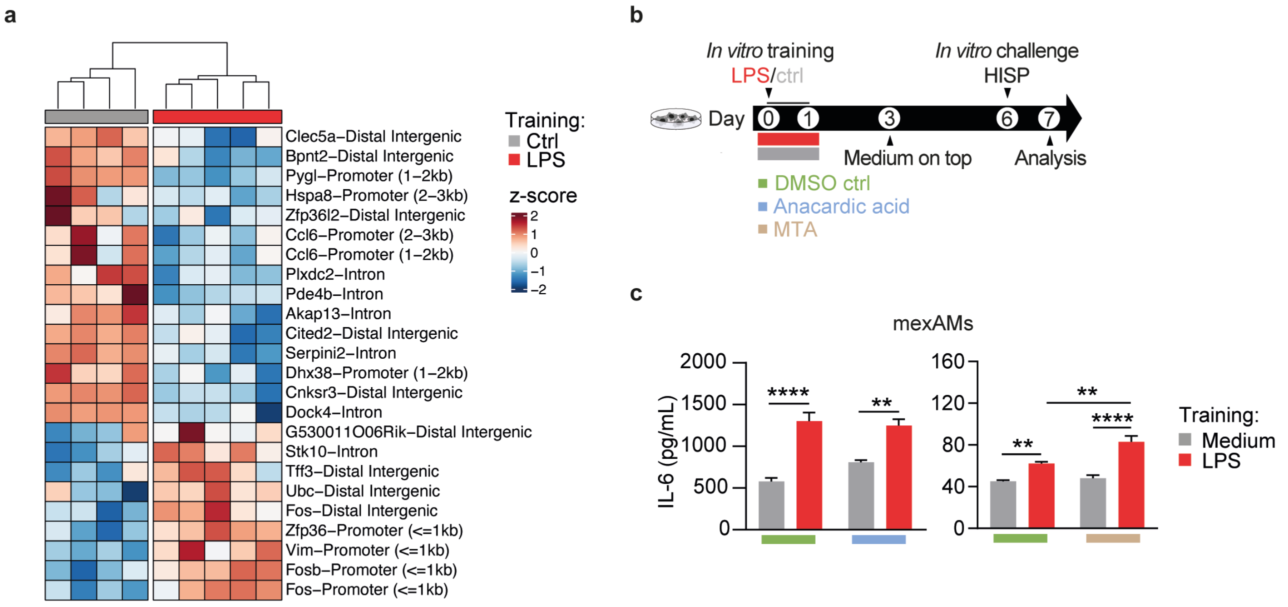

Fig. S4. AM ATAC-seq analysis and inhibition of epigenetic enzymes during mexAM training.
Refers to Fig. 3. **(a)** Heatmap displaying differentially accessible chromatin regions (adjusted p-value ≤ 0.05) of LPS-trained and control AMs. Cells were isolated and processed for ATAC-seq analysis six days after *in vivo* training. Raw counts were log_2-transformed, followed by z-score scaling. **(b)** Experimental setup for mexAM training with LPS or medium in presence of anacardic acid, 5'-deoxy-5'-methylthioadenosine (MTA) or DMSO, followed by *in vitro* HISP challenge (16 h) six days later. **(c)** IL-6 levels of mexAMs stimulated as described in **(b)**. Graphs show means + SEM of 4-5 technical replicates. Data are representative of two independent experiments. Statistical analysis: 2-way ANOVA (factor 1: training; factor 2: inhibitor). **p ≤ 0.01, ****p ≤ 0.0001.

Fig. S5

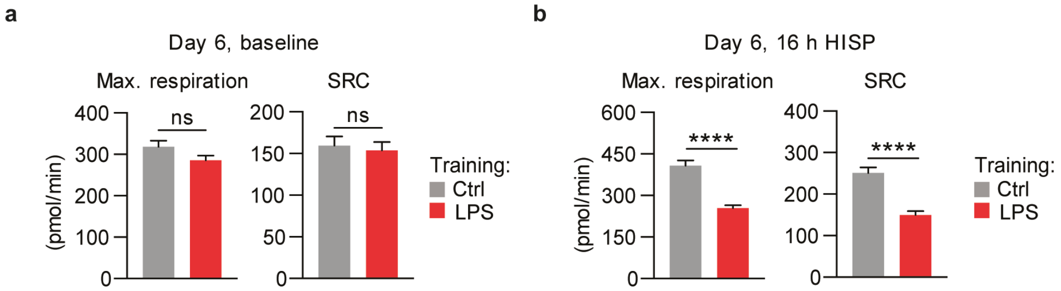

Fig. S5. Maximum and spare respiratory capacity of AMs on day six after *in vivo* training.

Refers to Fig. 4. **(a, b)** Maximum respiratory capacity (max. respiration) and spare respiratory capacity (SRC) of LPS-trained and control AMs on day six after *in vivo* training at baseline **(a)** and after *ex vivo* HISP challenge (16 h) **(b)**. Biological replicates (n=5-8) were pooled and seeded as technical replicates. Graphs show means + SEM of 10-11 technical replicates. Statistical analysis: student's t-test. ns, not significant. ****$p \leq 0.0001$.

Fig. S6

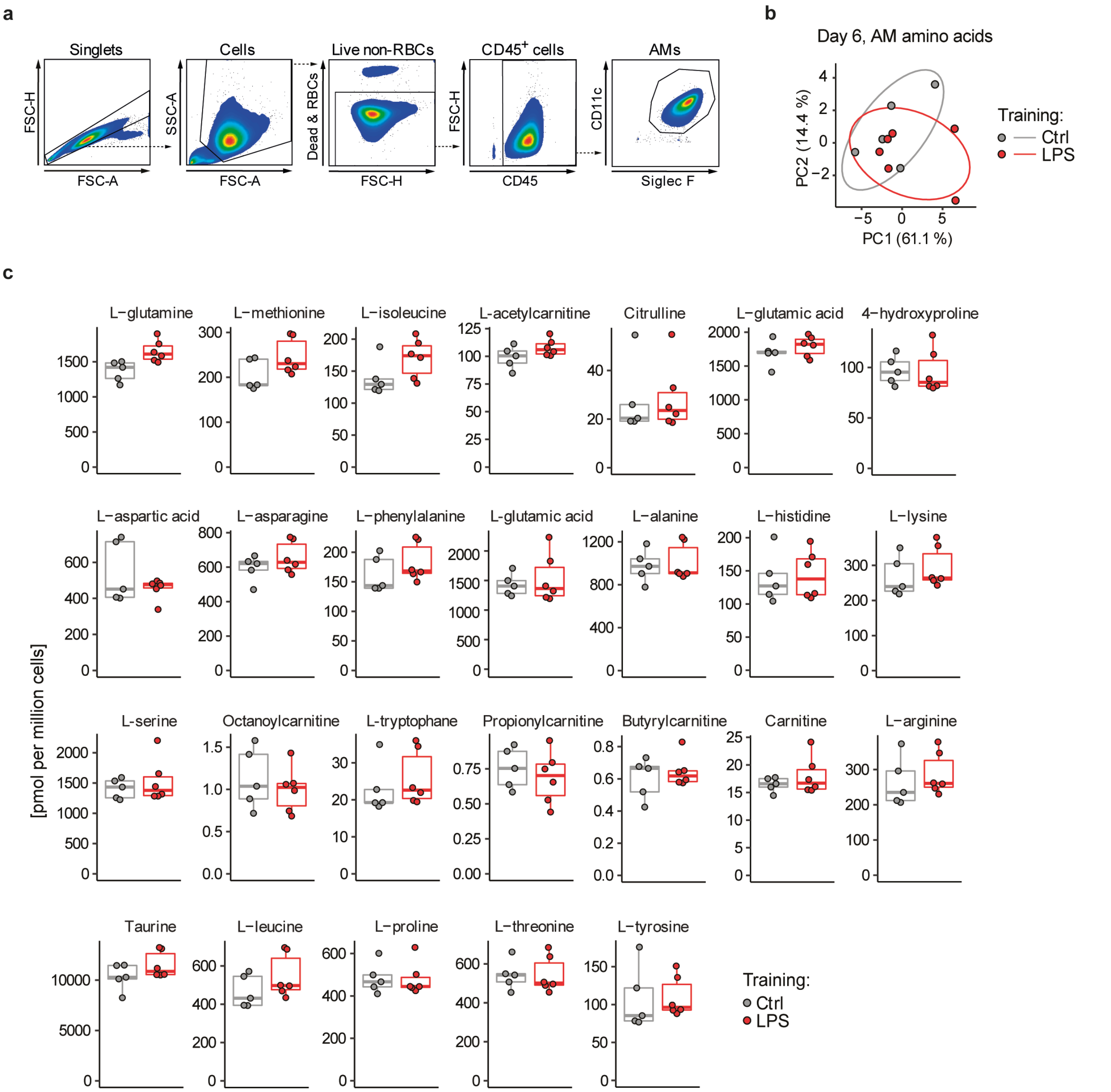

Fig. S6. AM amino acids six days after *in vivo* training.

Refers to Fig. 5. **(a)** Flow cytometry sorting strategy for isolation and LC-MS/MS analysis of CD11c^{+} Siglec F^{+} AMs on day six after *in vivo* training with LPS or saline. **(b, c)** Principal component analysis **(b)** and absolute concentration **(c)** of intracellular AM amino acids, detected six days after training. Graphs show medians and interquartile range of 5-6 biological replicates. Statistical analysis: student's t-test. Individual amino acids with p-values ≤ 0.05 and FDR ≤ 0.2 were considered statistically significant.

Fig. S7

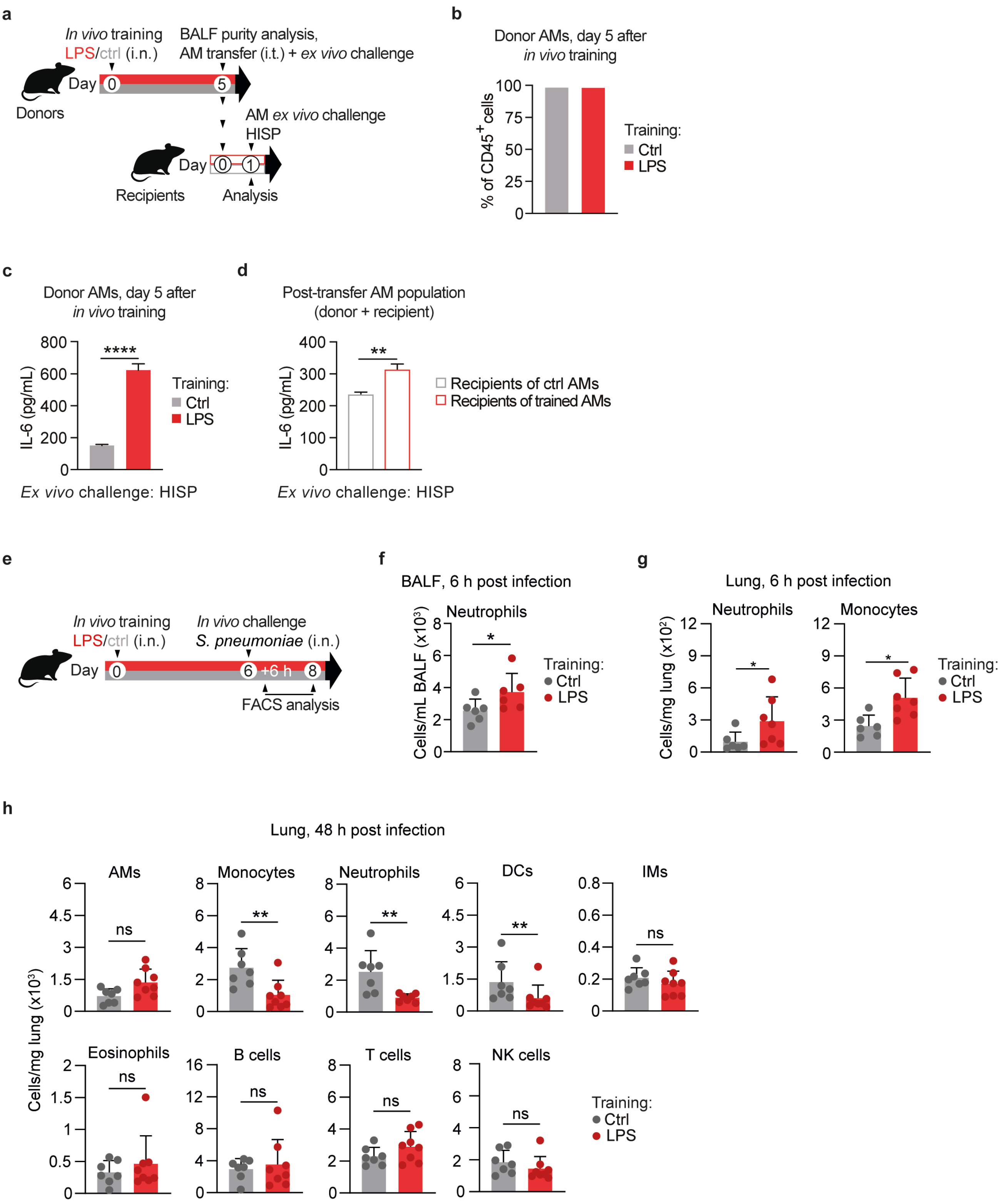
a
In vivo training
LPS/ctrl (i.n.)
BALF purity analysis,
AM transfer (i.t.) + ex vivo challenge
Day 0 5
Donors
AM ex vivo challenge
HISP
Day 0 1
Recipients
Analysis
b
Donor AMs, day 5 after
in vivo training
% of CD45+ cells
Training:
Ctrl
LPS
c
Donor AMs, day 5 after
in vivo training
IL-6 (pg/mL)
****
Training:
Ctrl
LPS
Ex vivo challenge: HISP
d
Post-transfer AM population
(donor + recipient)
IL-6 (pg/mL)
**
Recipients of ctrl AMs
Recipients of trained AMs
Ex vivo challenge: HISP
e
In vivo training
LPS/ctrl (i.n.)
In vivo challenge
S. pneumoniae (i.n.)
Day 0 6 +6 h 8
FACS analysis
f
BALF, 6 h post infection
Neutrophils
Cells/mL BALF (x10³)
*
Training:
Ctrl
LPS
g
Lung, 6 h post infection
Neutrophils
Monocytes
Cells/mg lung (x10²)
*
*
Training:
Ctrl
LPS
h
Lung, 48 h post infection
AMs
Monocytes
Neutrophils
DCs
IMs
Eosinophils
B cells
T cells
NK cells
Cells/mg lung (x10³)
ns
**
**
**
ns
ns
ns
ns
ns
Training:
Ctrl
LPS

Fig. S7. Assessment of AM engraftment and post-infection FACS analysis after *in vivo* training.

Refers to Fig. 6. **(a)** Experimental setup for adoptive transfer of trained and control AMs five days after *in vivo* training with LPS or saline. Donor BALF purity and successful establishment of donor AM memory were assessed by FACS analysis and *ex vivo* HISP challenge respectively. Donor cells were transferred intratracheally (i.t.) into naïve recipients. After 24 h, the chimeric AM population was isolated, followed by *ex vivo* HISP (16 h) challenge. **(b)** Frequencies of $CD11c^{+}$ Siglec F^{+} donor AMs, day five after *in vivo* training. Biological replicates (n=5) were pooled by group for purity analysis. **(c, d)** IL-6 levels of HISP-challenged donor AMs isolated on day five after *in vivo* training **(c)** or of HISP-challenged AMs isolated from recipients 24 h after adoptive transfer (i.e. comprising recipient and donor AMs) **(d)**. Biological replicates (n=5) were pooled by group and seeded as technical replicates. **(e)** Experimental setup for *in vivo* training with LPS/saline, followed by *S. pneumoniae infection* (*in vivo* challenge) six days later. BALF and/or lung tissue samples were analyzed by flow cytometry 6 h and 48 h after infection. **(f)** BALF neutrophil numbers, 6 h after infection. **(g)** Lung monocyte and neutrophil numbers, 6 h after infection. **(h)** Cellular composition of LPS-exposed and control lungs, 48 h after infection.

Data are representative of two independent experiments. Graphs show means + SEM of 5 technical replicates **(c, d)** or means + SD of 6-8 biological replicates **(f-h)**. Statistical analysis: student's t-test. ns, not significant. *p ≤ 0.05, **p ≤ 0.01, ****p ≤ 0.0001.

Table S1. Differentially expressed genes identified in AMs six days after *in vivo* training.
List of differentially expressed genes (DEGs) identified in LPS-trained and control AMs six days after *in vivo* training and 3 h after *ex vivo* incubation with medium (baseline). Genes are ordered by adjusted p-value (padj), from lowest to highest. DEGs with padj ≤ 0.1 were considered significant.

Table S2. Differentially expressed genes identified in HISP-challenged AMs six days after *in vivo* training.
List of differentially expressed genes (DEGs) identified in LPS-trained and control AMs six days after *in vivo* training and 3 h after *ex vivo* incubation with HISP. Genes are ordered by adjusted p-value (padj), from lowest to highest. DEGs with padj ≤ 0.1 were considered significant.

Table S3. Differentially accessible chromatin regions identified in AMs on day six after *in vivo* training.
List of differentially accessible regions (DARs) identified by ATAC-seq in LPS-trained and control AMs six days after *in vivo* training. Regions are ordered by adjusted p-value (padj), from lowest to highest. DARs with padj ≤ 0.05 were considered significant. Values in column “avlog2cpm” indicate average read counts of DARs expressed as log_2-transformed cpm (counts per million reads). TSS: transcription start site.

Table S4. Reagents, disposables and antibodies.
